# A simple reverse genetics method to generate recombinant coronaviruses

**DOI:** 10.15252/embr.202153820

**Published:** 2022-03-03

**Authors:** Julien Mélade, Géraldine Piorkowski, Franck Touret, Toscane Fourié, Jean‐Sélim Driouich, Maxime Cochin, Hawa Sophia Bouzidi, Bruno Coutard, Antoine Nougairède, Xavier de Lamballerie

**Affiliations:** ^1^ Unité des Virus Émergents (UVE: Aix‐Marseille Univ‐IRD 190‐Inserm 1207) Marseille France

**Keywords:** antivirals, *in vivo* experiment, reverse genetics, SARS‐CoV‐2, serology, Methods & Resources, Microbiology, Virology & Host Pathogen Interaction

## Abstract

Engineering recombinant viruses is a pre‐eminent tool for deciphering the biology of emerging viral pathogens such as the severe acute respiratory syndrome coronavirus 2 (SARS‐CoV‐2). However, the large size of coronavirus genomes renders the current reverse genetics methods challenging. Here, we describe a simple method based on “infectious subgenomic amplicons” (ISA) technology to generate recombinant infectious coronaviruses with no need for reconstruction of the complete genomic cDNA and apply this method to SARS‐CoV‐2 and also to the feline enteric coronavirus. In both cases we rescue wild‐type viruses with biological characteristics similar to original strains. Specific mutations and fluorescent red reporter genes can be readily incorporated into the SARS‐CoV‐2 genome enabling the generation of a genomic variants and fluorescent reporter strains for *in vivo* experiments, serological diagnosis, and antiviral assays. The swiftness and simplicity of the ISA method has the potential to facilitate the advance of coronavirus reverse genetics studies, to explore the molecular biological properties of the SARS‐CoV‐2 variants, and to accelerate the development of effective therapeutic reagents.

## Graphical abstract

Engineering recombinant viruses is a pre‐eminent tool for deciphering the biology of emerging viral pathogens. This study presents a simple and user‐friendly reverse genetic system to generate recombinant infectious coronaviruses.

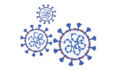

## Introduction

The order *Nidovirales* represents a large group of single‐stranded positive‐sense RNA viruses ((+) ssRNA) characterized by the size of their genomes, which are the largest (~30 kilobases) among the RNA viruses (Gorbalenya *et al*, 2006). This order is subdivided into nine suborders including *Cornidovirineae* in which is found the *Coronaviridae* family. Coronaviruses (CoVs) can infect a wide range of hosts including humans, domestic, and wild animals (Masters, 2006).

The severe acute respiratory syndrome coronavirus 2 (SARS‐CoV‐2; genus: *Betacoronavirus*) which emerged in 2019 in Wuhan (Zhu *et al*, 2020), is responsible for COVID‐19, a human disease that, in a proportion of patients, has been associated with severe pneumonia leading to respiratory distress and in many cases to death (Zhou *et al*, 2020). SARS‐CoV‐2 has spread worldwide with more than 67 million people being infected by the end of 2020. During the course of the pandemic, a European variant carrying an amino acid change in the spike protein (D614G) rapidly dispersed worldwide and became the most prevalent and dominant pandemic strain (Korber *et al*, 2020). Later in 2020, other variants were detected in Denmark (Kmec, 2020), South East England (Wise, 2020), Brazil (Voloch *et al*, 2020), and South Africa (Tegally *et al*, 2020). The emergence of these variants raised questions concerning the possibility of viral escape from the immune response induced following either primary infection, vaccination, or therapeutics applied as convalescent plasma (Weisblum *et al*, 2020). The ongoing emergence of variants, their circulation, and the genetic diversity observed in CoV populations highlight the need for convenient molecular tools to study viral evolution, replication, and pathogenesis and to enable the development of appropriate health control countermeasures.

Reverse genetic methods enable the engineering of wild‐type or genetically modified CoVs and thus can contribute to deciphering biological properties of human or animal viruses (Almazán *et al*, 2014; Stobart & Moore, 2014). In addition, they can be used to expedite antiviral screening for the selection and characterization of small antiviral molecules or therapeutic antibodies (Yang *et al*, 2014). In the case of CoVs, rescue of infectious viruses can be achieved by the transfection of infectious cDNA using vaccinia virus vectors or bacterial artificial chromosomes (Almazan *et al*, 2000; Yount *et al*, 2000; Almazán *et al*, 2014; Thiel *et al*, 2017; Rihn *et al*, 2021). Alternatively, *in vitro* or “in‐yeast” viral genome assembly followed by *in vitro* RNA production can lead to rescued viruses by transfection of full‐length cDNA in cells (Thi Nhu Thao *et al*, 2020; Xie *et al*, 2020a). Due to the complexity and large size of CoV genomes, the assembly and modification of full‐length genomic RNA remains laborious, technically difficult to reproduce and time‐consuming (e.g., toxicity of clones, difficulty in constructing precise full‐length 30‐kb RNA transcripts *in vitro*).

The Infectious Subgenomic Amplicons (ISA) method is a simple and rapid bacteria‐free method that has been developed in recent years for viruses with relatively short (+) ssRNA genomes, including members of the *Flaviviridae*, *Togaviridae,* and *Picornaviridae* families (Aubry *et al*, 2014, 2015; Atieh *et al*, 2016, 2017; Touret *et al*, 2019). With the ISA method, wild‐type and genetically modified infectious viruses can be recovered within days, whereas conventional reverse genetic systems require additional cloning steps or *in vitro* manipulation of the RNA molecules. The ISA method is based on the simple transfection of overlapping subgenomic DNA fragments, encompassing the entire virus genome into permissive cells. DNA recombination and production of full‐length viral genomic RNA, under transcription signals, are accomplished by the cellular machinery.

In the current study, we described a user‐friendly and simple reverse genetics system to generate recombinant coronaviruses. We rescued the wild‐type European variant of SARS‐CoV‐2 and the feline enteric coronavirus (FeCoV), an ubiquitous veterinary pathogen commonly circulating in felid populations and responsible for common enteritis (Pedersen *et al*, 2008) to severe systemic disease called feline infectious peritonitis (FIP) (Pedersen, 2009). We derived the original D614 coding sequence of the Wuhan SARS‐CoV‐2 by mutagenesis and added an mCherry fluorescent reporter gene. The characterization of each rescued strain, performed *in vivo* using a golden Syrian hamster model for SARS‐CoV‐2, plus seroneutralization tests and antiviral assays were conducted using the mCherry fluorescent SARS‐CoV‐2 strain. Our results demonstrate the suitability of the strategy to study biological properties of viruses engineered using the ISA method.

## Results & Discussion

### General strategy for *de novo* production of CoVs

**Figure 1:**
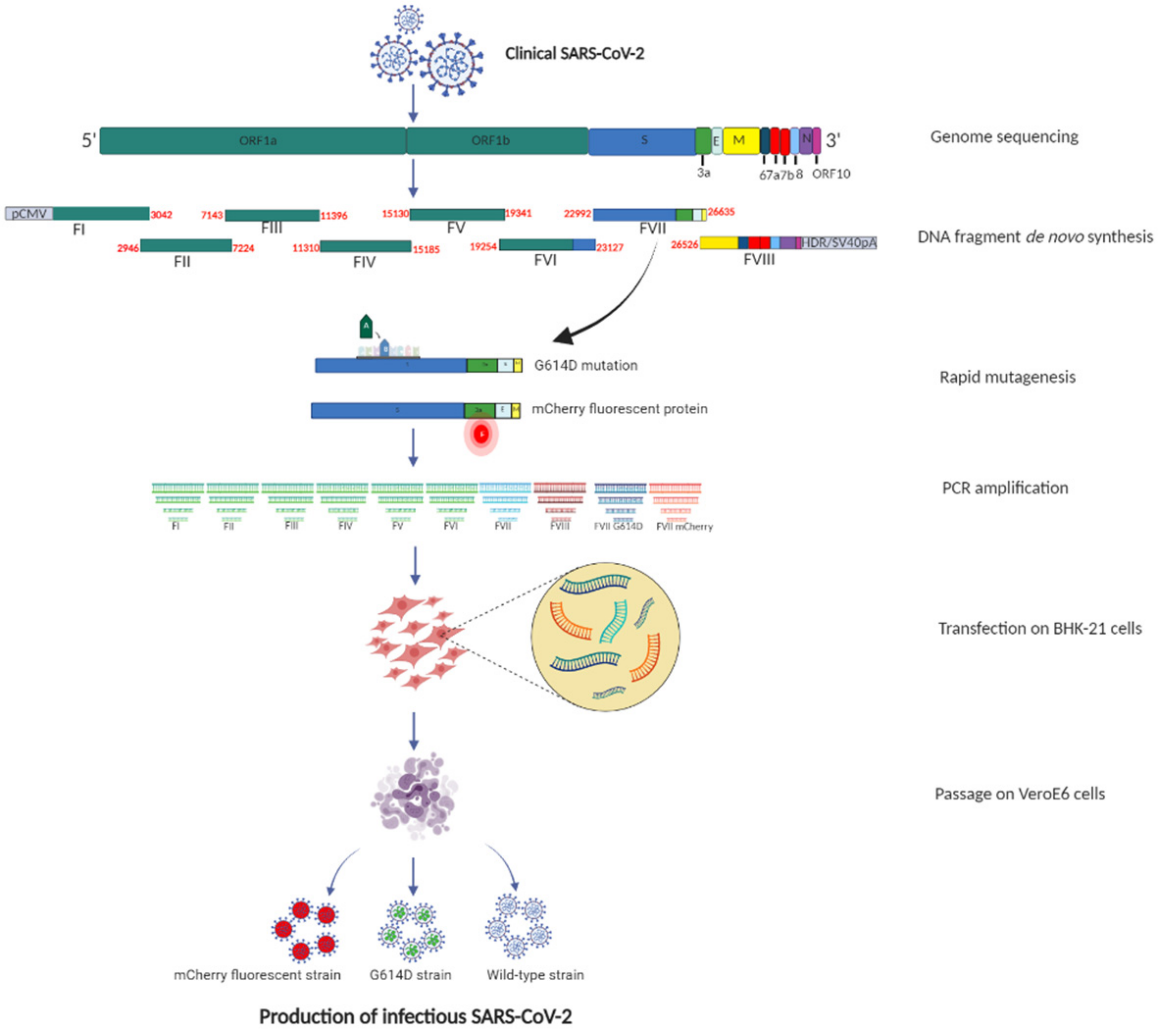
The ISA method to rescue SARS‐CoV‐2 SARS‐CoV‐2 complete genome sequence was used to design eight overlapping subgenomic viral fragments covering the complete genome. Positions on the genome (in nucleotide) are indicated in bold red. This figure was created with BioRender.com.

From each full‐length genome sequence, eight overlapping subgenomic DNA fragments with an average size of 3,900 nucleotides were designed and *de novo* synthesized (Fig 1). During *de novo* synthesis, the human cytomegalovirus promoter (pCMV) was inserted upstream from the first fragment. Additionally, the sequence of the hepatitis delta virus ribozyme followed by the simian virus 40 polyadenylation signal (HDR/SV40pA) were added at the 3’ end of the last fragment. After transfection and fragment recombination, the pCMV initiates the DNA transcription to produce mRNA by utilizing RNA polymerase II. Following this, the HDR/SV40pA facilitates transcription termination with RNA maturation, splicing, and poly‐adenylation. This ensures the production of infectious viral particles. These synthetic subgenomic viral fragments were used as templates for PCR amplification and transfected into permissive cells. Five days after transfection, supernatants were passaged once on infection‐competent cells and incubated for 2 days. A second passage was performed under the same conditions. Infectious viral particles were successfully obtained after two passages as confirmed by (i) the observation of a cytopathic effect, (ii) the measurement of the viral RNA load in cell supernatant medium using a real‐time RT‐qPCR assay and TCID_50_ assays, and (iii) the measurement of the infectious viral load in cell supernatant medium using TCID_50_ assays. Each experiment was tested in three distinct wells. Additionally, for clinical and recombinant SARS‐CoV‐2, a subgenomic viral RNA quantification was performed by RT‐qPCR and the spike, nucleocaspid, membrane, and the envelope proteins were amplified by RT‐PCR.

### Generation of wild‐type CoVs

**Figure EV1:**
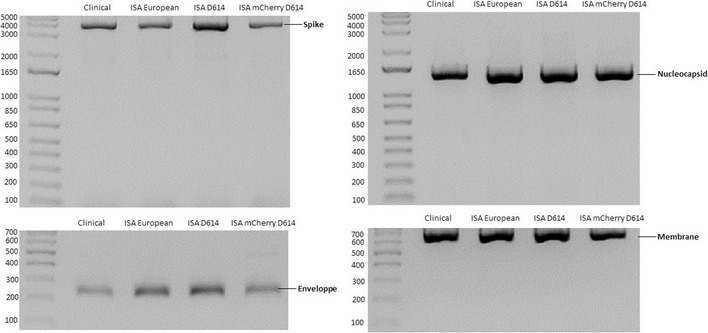
The spike, nucleocapsid, membrane, and envelope proteins of clinical and recombinant SARS‐CoV‐2 after two passages in VeroE6 were amplified by RT‐PCR from clarified supernatant medium

**Figure 2:**
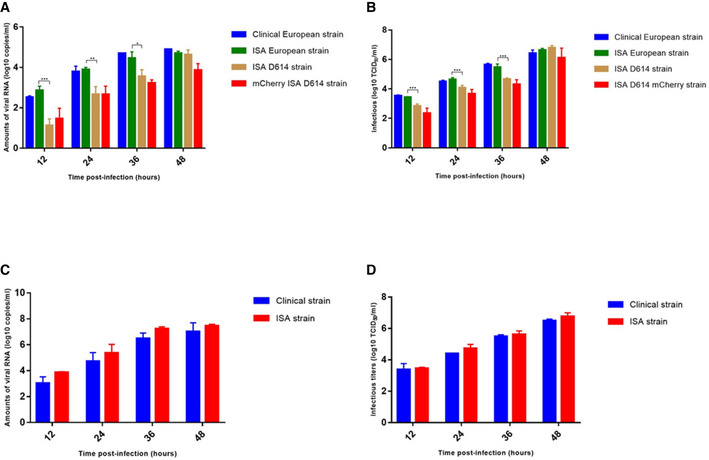
Virus replication kinetics of clinical and ISA strains A, BAn moi of 0.001 was used to infect VeroE6 with rescued or clinical SARS‐CoV‐2 (A, B).C, DAn moi of 0.01 was used to infect FeA cells with rescued or clinical FeCoV (C, D). Data information: Data are represented as mean ± SD (indicated by the error bars). Each experiment was performed in technical triplicates (*N* = 3). Exploratory analyses were performed using a two‐way ANOVA for multiple comparisons with Sidak’s multiple comparisons test. Statistical comparisons were performed between SARS‐CoV‐2 clinical European vs ISA European strains, ISA European vs ISA D614 strains, ISA D614 vs mCherry D614 strains, and between FeCoV clinical vs ISA strains. Only *P*‐values ≤ 0.05 were indicated by a * symbol. In other cases, *P*‐values > 0.05 were considered as not significant and were not displayed on the graph. ***, **, and * symbols indicate that the average value for the ISA D614 strain is significantly different from that of the ISA European strain with *P*‐values < 0. 0001, < 001, and ≤ 0.05, respectively.

Firstly, wild‐type infectious particles of the European SARS‐CoV‐2 were recovered at the second passage on VeroE6 cells, when extensive cytopathic effect (cpe) was observed from 2 days post‐infection (dpi) similar to the clinical strain. The production of infectious particles was confirmed by the average genomic viral RNA load (5.5 ± 0.4 log_10_ RNA copies per ml), infectious viral load (5.5 ± 0.4 log_10_ TCID_50_ per ml; Table 1) detected in the cell culture supernatant medium, and subgenomic RNA (7.6 ± 0.1 log_10_ sgRNA copies per ml; Appendix Table S1) in the cell lysate at 2 dpi in the three tested wells. RT‐PCR amplification of the spike, nucleocaspid, membrane, and the envelope proteins of clinical and ISA European SARS‐CoV‐2 after two passages on VeroE6 showed no difference (Fig EV1). Sequencing of the whole genome after two passages in VeroE6 cells indicated one single synonymous majority mutation (position 18,538, C → A) and one non‐synonymous minority mutation (position 27,444, C → T, frequency: 10%; Table 2). Replication kinetics were performed to compare the replicative fitness of the clinical and rescued viruses. Clinical and ISA strains showed similar replication kinetics and no significant difference in viral RNA loads and infectious load was observed post‐infection (pi; *N* = 3; Two‐way ANOVA*; P* > 0.05; Fig 2A and B).

**Table 1:** Phenotypic characterization of rescued SARS‐CoV‐2 and FeCoV.

		Cell line		Cpe	Amount of viral RNA	Infectious titers
		Transfection	Passage			
SARS‐CoV‐2	Clinical European	–	VeroE6	Yes	7.1 ± 0.2	7.6 ± 0.1
	ISA European	BHK‐21 + VeroE6	VeroE6	Yes	5.5 ± 0.4	5.5 ± 0.4
	ISA D614	BHK‐21 + VeroE6	VeroE6	Yes	6.8 ± 0.5	6.0 ± 0.2
	mCherry ISA D614	BHK‐21 + VeroE6	VeroE6	Yes	5.6 ± 0.2	5.8 ± 0.4
FeCoV	Clinical	–	FeA	Yes	7.6 ± 0.2	7.2 ± 0.2
	ISA	BHK‐21 + FeA	FeA	Yes	6.7 ± 0.5	5.8 ± 0.6

For SARS‐CoV‐2 and FeCoV clinical and ISA strains, cell lines used for transfection and passage, the presence or absence of cytopathic effect (cpe), quantification of the virus RNA by real‐time RT‐qPCR and infectious titers in cell supernatant media (TCID_50_ assay) after two passages were summarized. Each experiment was performed in triplicates (*N* = 3). The infectious TCID_50_ titer was expressed as log_10_ TCID_50_/ml and the amount of RNA copies was expressed as log_10_ copies/ml.

**Table 2:** Genotypic characterization.

	Majority mutation			Minority mutation			
	Position	Nt change	AA change	Position	Nt change	AA change	Frequency (%)
ISA European	18,538	C → A	n.d				
				27,444	C → T	T → I	10
ISA D614	n.d	n.d	n.d				
				14,290	C → T	n.d	8
				16,110	A → C	D → A	17
				28,663	C → T	T → I	5
ISA mCherry	1,871	A → G	n.d				
				283	G → T	n.d	10
				7,789	C → T	S → F	11
ISA FeCoV	n.d	n.d	n.d	n.d			

Complete genome analyses of SARS‐CoV‐2 and FeCoV obtained by the ISA method after two passages. Characteristics of majority (mutations with frequencies >50% in sequencing reads) and minority mutations (mutations with frequencies between 5 and 50% in sequencing reads) are described according to their position, region, nucleotide and amino acid change. Nt: Nucleotide; AA: amino acid; n.d: not detected.

**Figure EV2:**
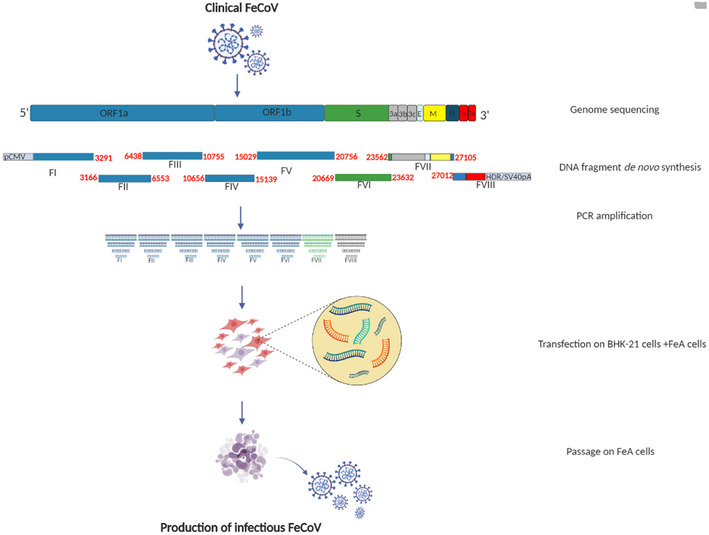
The ISA method to rescue FeCoV FeCoV complete genome sequence was used to design eight overlapping subgenomic viral fragments covering the full genome. Positions on the genome (in nucleotide) are indicated in bold red. This figure was created with BioRender.com.

We further evaluated the robustness of the ISA method to reconstruct a well‐known and widespread felid coronavirus, FeCoV (Fig EV2). After two passages of cell supernatant on FeA cells, extensive cpe was observed. The presence of rescued viral particles was confirmed by the average viral RNA load and infectious viral loads at 2 dpi in the cell culture supernatant medium in the three tested wells (6.7 ± 0.5 log_10_ RNA copies per ml and 5.8 ± 0.6 log_10_ TCID_50_ per ml, respectively; Table 1). No mutations were detected along the full genome sequence after two passages on FeA cells (Table 2). Comparative replication kinetics at an moi of 0.01 did not show significant differences in virus yield from 12 h until the endpoint (2 dpi; *N* = 3; Two‐way ANOVA; *P* > 0.05; Fig 2C and D).

### Generation of the D614 SARS‐CoV‐2 strain

Early in the SARS‐CoV‐2 pandemic, several mutations were observed when comparing the original strain from Wuhan and the strain circulating in Europe. Among these mutations, the D614G on the spike protein sequence was suspected to contribute to changing the fitness of the virus (Hou *et al*, 2020a; Plante *et al*, 2020). To generate the spike protein D614 coding sequence in the ISA European strain, we substituted the Gly at position 614 of the spike protein sequence in fragment number 7 with an Asp and conducted the ISA method using this *de novo* synthesized modified DNA fragment (Fig 1). After 5 days post‐transfection on BHK‐21 and two passages on VeroE6 cells, infectious particles of the D614 SARS‐CoV‐2 strain were obtained and confirmed by the presence of cpe, genomic viral RNA load, and infectious loads in the cell culture supernatant medium (Table 1) or cell lysate (Appendix Table S6) at 2 dpi. No difference in the spike, nucleocaspid, membrane, and the envelope proteins amplification between both strains were recorded (Fig EV1). Sequencing of the whole viral genome after two passages in VeroE6 cells indicated no majority mutation in the consensus sequence and three minority mutations (position 14,290, C → T, frequency: 8%; position 16,110, A → C, frequency: 17%; position 28,663, C → T, frequency: 5%; Table 2). Every 12 h pi, the viral RNA load and infectious load in the supernatant medium of infected Vero E6 cells were recorded and analyzed. Interestingly, significant differences were observed in early collections, particularly at 12 h and 24 h pi where viral RNA loads for the ISA D614 strain were 1.1 ± 0.3 and 2.7 ± 0.3 log_10_ RNA copies per ml respectively, and 3.1 ± 0.2 and 3.9 ± 0.2 log_10_ RNA copies per ml respectively, for the ISA European strain in the three tested wells (Fig 2A; *N* = 3; Two‐way ANOVA*; P* < 0.05). Similar observations were recorded in the infectious viral loads (Fig 2B). At the endpoint, no significant difference was observed (48 h pi) between viral RNA loads and infectious loads for ISA D614 and European strains (4.6 ± 0.2 and 4.7 ± 0.1 log_10_ RNA copies per ml, respectively; 6.2 ± 0.3 and 6.6 ± 0.1 log_10_ TCID_50_ per ml, respectively; *N* = 3; two‐way ANOVA*; P* > 0.05).

### Generation of a fluorescent SARS‐CoV‐2

**Figure 3:**
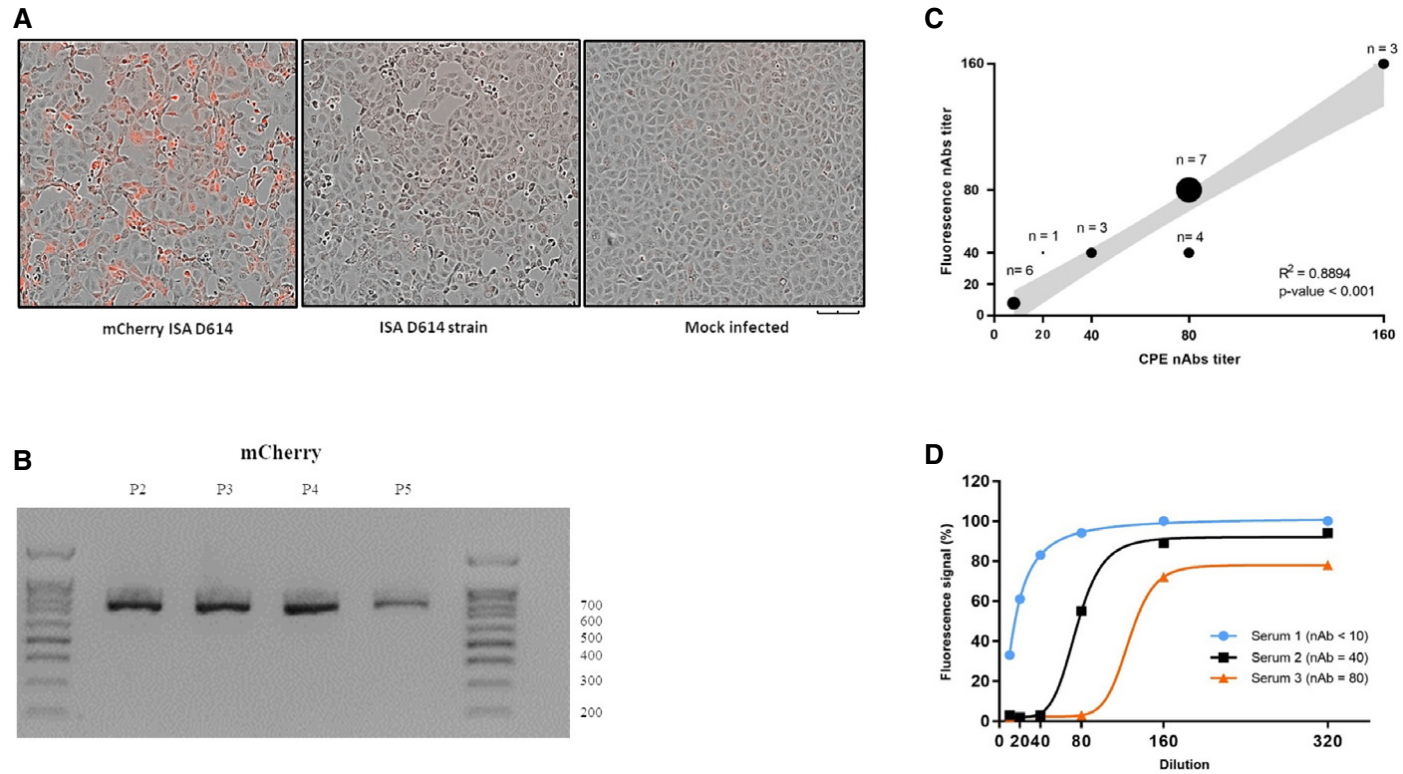
Fluorescence microscopy analysis and correlation between titers of neutralizing antibodies (nAb) using ISA D614 and mCherry D614 SARS‐CoV‐2 strains Vero E6 cells were infected with an moi of 0.05 with the fluorescent mCherry D614 strains, wild‐type ISA D614 or mock infected. Pictures were taken at 48 h pi (20×). Scale bar, 100 µm.The mCherry reporter gene in the ISA mCherry D614 on VeroE6 cells supernatant medium at passages 2 to 5 (p2, p3, p4, and p5) was RT‐PCR amplified and analyzed using gel electrophoresis.Twenty‐four human sera were then two‐fold diluted and incubated with the ISA D614 and mCherry D614 strains and nAb titers were recorded at 5 days dpi. nAb titers were defined as the highest dilution that inhibited the production of distinct cpe with the ISA D614 SARS‐CoV‐2 or fluorescence with the fluorescent mCherry D614 SARS‐CoV‐2. Each black dot represents results from a given number of sera. Statistical analyses were performed using univariate linear regression. The error band (in grey) represents the 95% confidence interval of the regression line. The Pearson correlation coefficient (*R* ^2^) and *P*‐value analyses are shown.Representative neutralizing curves of the nAb fluorescence‐based assay. The four‐parameter dose–response curve was fitted using the nonlinear regression method and nAbs were calculated in the software Prism 7.0. For negative serum samples, an arbitrary value of 10 was assigned (detection threshold for both methods).

We next engineered a D614 SARS‐CoV‐2 strain containing an mCherry monomeric red fluorescent protein downstream to the regulatory sequence of the ORF3a (position 25,392–26,221). The hypothetically dispensable 3a region (Yount *et al*, 2005) was thus removed and replaced by the mCherry protein sequence and the modified DNA fragment for the ISA procedure was *de novo* synthesized (Fig 1). Following the ISA procedure, 5 days post‐transfection on BHK‐21 or 2 dpi on VeroE6 cells, infectious fluorescent mCherry D614 SARS‐CoV‐2 strain was recovered and similar cpe was observed at 48 h pi. At an moi of 0.05, red fluorescence was readily detectable in the infected cells at 48 h pi (Fig 3A) in comparison with the ISA D614 infected cells or in the mock infected cell negative control. The genomic viral RNA load and infectious loads detected at 2 dpi in Vero cell supernatant medium are indicated in Table 1. We observed one synonymous majority mutation (position 1,871, A → G) and one silent (position 283, G → T, frequency: 10%) and non‐silent minority mutation (position 7,789, C → T, frequency: 11%) in the complete genome sequence of the mCherry D614 SARS‐CoV‐2 after two passages in VeroE6 cells (Table 2). The mCherry D614 strain replicative fitness was assessed using the same conditions as previously described and compared with the ISA D614 SARS‐CoV‐2 strain. Replicative fitness was similar between fluorescent mCherry D614 and ISA D614 strains and no significant difference in genomic viral RNA load between both strains was recorded at any time pi (Fig 2A and B). After five passages on VeroE6 cells, the presence of the mCherry reporter gene was confirmed by RT‐PCR (Fig 3B) and NGS sequencing. When compared with the original synthesized fragment, no mutations or deletions were identified.

### Development and evaluation of neutralization and antiviral assays using the fluorescent mCherry SARS‐CoV‐2

A seroneutralization assay was established by exploiting the fluorescence of the mCherry D614 SARS‐CoV‐2 for the endpoint readout and this was compared with a reference procedure (Gallian *et al*, 2020) using the D614 strain, relying on the manual detection of cpe after image recording of the culture wells. Twenty‐three human sera, collected during the COVID‐19 pandemic, were tested for neutralization in the assay. Qualitatively, all the sera showing neutralization (18 out of 23) in the standard procedure performed equally well in the fluorescent procedure (Appendix Table S2) and all the negative sera in the cpe‐based method were also negative in the fluorescence‐based tests. Titration of each serum using both methods indicated that seroneutralization titers were significantly correlated throughout the entire range of dilutions (*P* < 0.0001) with a Pearson correlation coefficient (R^2^) of 0.8894 (Fig 3C). A classical dose‐dependent increase in the fluorescence was observed (Fig 3D) demonstrating that the mCherry D614 SARS‐CoV‐2 can be used for neutralization assays.

**Figure 4:**
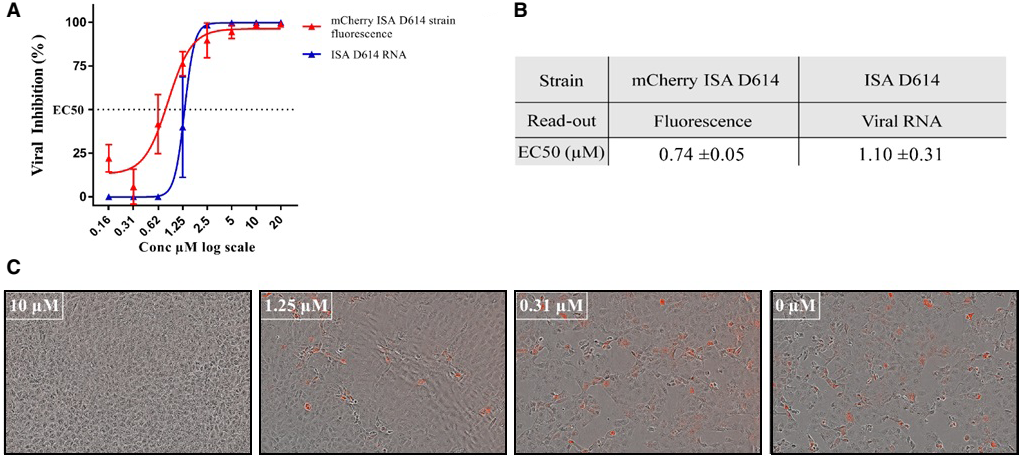
Remdesivir antiviral activity on SARS‐CoV‐2 in VeroE6 cells Dose–response curve for the ISA D614 and for the mCherry D614 strains obtained by fluorescence or viral RNA measurement in VeroE6 cells from one representative experiment. Data are represented as mean ± SD (indicated by the error bars). Each experiment was performed in technical triplicates (*N* = 3).Table of EC_50_ values obtained for the two different strains from two technical replicates and their respective mean ± SD.Fluorescence of the SARS‐CoV‐2 mCherry in VeroE6 cells with different Remdesivir concentration. Scale bar, 200 µm. Data information: EC_50_: 50% inhibition, Remdesivir concentrations are presented in log scale for logarithmic interpolation. Dose–response curves were generated using GraphPad Prism software version 7.0 (https://graphpad‐prism.software.informer.com/7.0/) with a four‐parameter linear regression.

We next evaluated the mCherry D614 SARS‐CoV‐2 strain for suitability in an antiviral assay. Remdesivir was used as a reference compound known to inhibit the viral replication *in vitro* at the µM level (Touret *et al*, 2020; Wang *et al*, 2020). The 50% effective concentration (EC_50_) was determined by monitoring the fluorescence in the presence of decreasing concentrations of Remdesivir (Fig 4A). The EC_50_ was compared with values obtained for the ISA D614 strains using a standard procedure relying on the quantification of the viral RNA yields (Wang *et al*, 2020). The EC_50_ obtained by measuring the fluorescence of the mCherry D614 strain was in agreement with the EC_50_ obtained by the standard method (0.74 ± 0.05 vs 1 ± 0.31; Fig 4B) as well as with the EC_50_ value reported using the same SARS‐COV‐2 strain (Wang *et al*, 2020). Moreover, these values are also perfectly coherent with the observations where fluorescence and cpe inhibition were observed at 1.3 µM and 10 µM but not at 0.3 µM of Remdesivir concentration (Fig 4C).

### Infection of Syrian hamsters with ISA viruses

**Figure 5:**
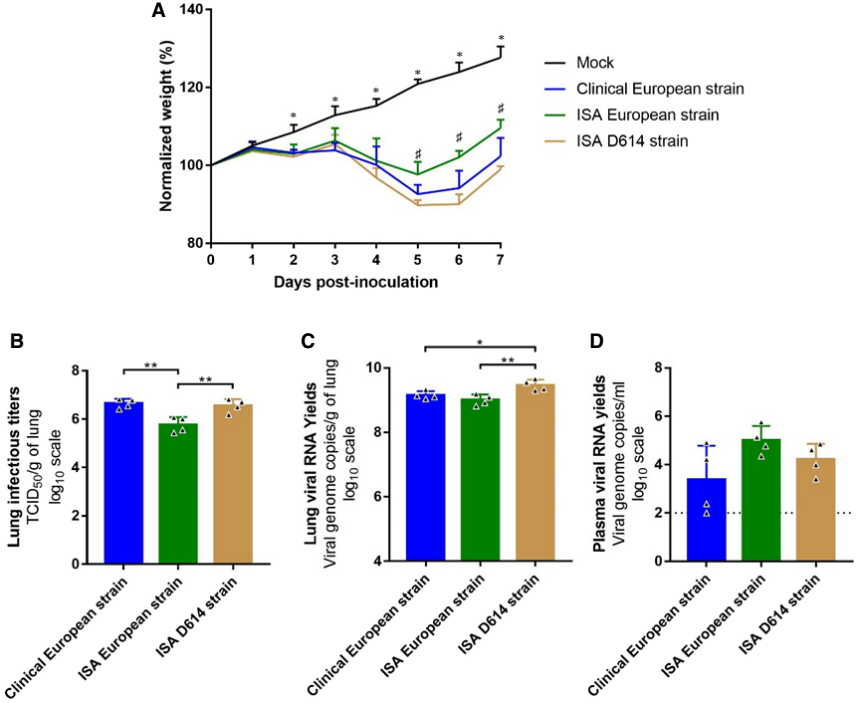
Body weight changes and viral replication in tissues after infection by SARS‐CoV‐2 in Syrian gold hamsters Groups of four hamsters were intranasally infected with 10^3^ TCID_50_ of clinical European, ISA European or ISA D614 strain. After 3 dpi, viral RNA loads and infectious viral loads were assessed in lung and plasma. Clinical course of the disease. Normalized weight at day n was calculated as follows: % of initial weight of the animal at day *n*.Lung infectious titers (measured using a TCID_50_ assay) expressed in TCID_50/_g of lung.Lung viral RNA yields (measured using an RT‐qPCR assay) expressed in virus genome copy/g of lung.Plasma viral RNA loads (measured using an RT‐qPCR assay) expressed in viral genome copies/ml of plasma. Data information: Data are represented as mean ± SD (indicated by the error bars). For all graph, each experiment was performed in four biological replicates (*N* = 4). Exploratory analysis was performed using a two‐way ANOVA with a Sidak’s test correction. Only *P*‐values ≤ 0.05 were indicated by a * or ^#^ symbol. For graph a, significant differences between mock‐infected animals and clinical/ISA European/ISA D614 SARS‐CoV‐2‐infected animals were indicated by a *; significant differences between clinical European/ISA D614 SARS‐CoV‐2‐infected animals and ISA European SARS‐CoV‐2 strain‐infected animals were indicated by a ^#^. For graph (B), (C), and (D), ** and * symbols indicate significant difference with a *P*‐value ranging between 0.001–0.01 and 0.01–0.05, respectively (details in Appendix Tables S8 and S9). In other cases, *P*‐values > 0.05 were considered as not significant and were not displayed on the graph.

A hamster model was used to study the clinical and virological properties of clinical and ISA‐constructed SARS‐CoV‐2 strains. Groups of four animals were infected by intranasal inoculation of 10^3^ TCID_50_ of viruses. Clinical monitoring of animals infected by clinical and ISA SARS‐CoV‐2 showed a significant weight loss from 2 dpi when compared to animals inoculated with 0.9% sodium chloride solution (Two‐way ANOVA; *P* ≤ 0.01). From 0 to 4 dpi, infected animals expressed similar normalized weights (Two‐way ANOVA; *P* ≥ 0.05). However, from 5 to 7 dpi, animals infected with the clinical European strain or the ISA D614 strain expressed a greater weight loss than those infected with the ISA European SARS‐CoV‐2 (Two‐way ANOVA; *P* ≤ 0.05; Fig 5A).

For each virus strain, infection and replication were confirmed as infectious virus was recovered from lungs and viral RNA was detected in lungs and plasma at 3 dpi. Analysis of virus replication in clarified lung homogenates revealed that mean infectious titers (measured using TCID_50_ assay) were 6.6, 5.8, and 6.6 log_10_ TCID_50_/g of lung, for animals infected with clinical European, ISA European, and ISA D614 strains, respectively. Infectious titers of virus recovered from hamsters infected by the ISA D614 strain were significantly lower than those rescued from hamsters infected with the clinical or the ISA European strains (*P* ≤ 0.01; Fig 5B). Mean viral RNA yields (measured using quantitative real‐time RT‐PCR assay) were 9.2, 9, and 9.5 log_10_ copies/g of lung for animals infected with clinical European, ISA European, and ISA D614 strains, respectively. Viral RNA yields in lungs of hamsters infected by the ISA D614 strain were significantly higher than those of hamsters infected with the clinical European or the ISA European strains (*P* ≤ 0.05; Fig 5C). Analysis of virus replication in plasmas revealed no significant difference between the three strains (*P* ≥ 0.05). Mean viral RNA yields (measured using quantitative real time RT‐PCR assay) were 3.4, 5.0, and 4.2 log_10_ copies/ml, respectively, for animals infected with clinical European, ISA European, and ISA D614 strains (Fig 5D). No change in the genetic sequences was observed in each ISA virus.

Reverse genetics methods are valuable modern tools to decipher the biological properties of human and animal coronaviruses, to define the mechanisms that underlie viral emergence and adaptation to the host, and to develop therapeutic strategies. Although coronaviruses have the largest genomes of known human RNA viral pathogens, several techniques were developed for the production of infectious clones before the emergence of the COVID‐19 pandemic and they have been successfully adapted to the study of SARS‐CoV‐2 (Thi Nhu Thao *et al*, 2020; Xie *et al*, 2020a; Rihn *et al*, 2021). These techniques require the cloning of the full genome cDNA in a plasmid, a step that is bypassed using the ISA method relying on simultaneous *ex vivo* recombination and transcription of viral RNA. Thus, no restriction sites or other genomic modifications are required. The method was originally developed for a variety of viruses with relatively short positive‐stranded RNA genomes (i.e., < 15,000 nucleotides) such as flaviviruses, alphaviruses, or enteroviruses (Aubry *et al*, 2014, 2015; Atieh *et al*, 2017; Touret *et al*, 2019). For these viruses, usually three overlapping subgenomic DNA fragments is sufficient to encompass the entire genomic cDNA and to flank the 5′ and 3′ ends with a transcription start and a ribozyme/polyA signal, respectively. In the case of the much larger CoV genome, we found that by using eight overlapping subgenomic fragments, the ISA method could be used to produce either wild‐type or genetically modified CoVs.

However, as far as we are aware, recombination *in cellulo* of such a high number of fragments has not previously been evaluated. The objective, therefore, was to assess whether or not we had reached the technical limitations of the ISA method. Full‐length cDNA reconstitution implies two constraints: that cells generating infectious RNA should receive all fragments covering the entire virus genome, and that all fragments recombine together. An obvious consequence of this procedure is that the probability for an individual cell to receive all subgenomic fragments simultaneously upon transfection decreases when the number of fragments increases. Accordingly, in order to integrate the stochastic aspect of viral genesis in the ISA process, transfection/infection experiments can be conveniently performed in 96‐well tissue culture plates with a total of ~30 µg of mixed PCR fragments obtained from PCR reaction for each fragment. It is well established that deletions can occur in the spike protein of SARS‐CoV‐2 and especially in the furin cleavage site (Liu *et al*, 2020). Here, the infectious supernatant medium of each rescued strain after two passages on VeroE6 cells was additionally passaged three more times to determine whether or not additional deletions in the complete genome arose, particularly in the spike protein, and indicated a conserved genomic structure. However, the common use of Vero E6 TMPRSS2 in the future would in any case avoid such deletions arising (Hoffmann *et al*, 2020; Matsuyama *et al*, 2020).

Mutagenesis within the original sequence fragments can be accomplished using subgenomic sequences without jeopardizing subsequent genomic assembly. The resulting product is immediately ready for the ISA procedure which can be exploited to decipher the mechanisms involved in virus evolution, transmission, pathogenesis, and virus/host interactions (Wang *et al*, 2008; Eckerle *et al*, 2010; Menachery *et al*, 2018; Muth *et al*, 2018; Coutard *et al*, 2020; Hoffmann *et al*, 2020; Korber *et al*, 2020). For the proof of concept, we firstly designed and *de novo* mutated a modified synthetic fragment to generate the D614 ISA strain with the mutation in the spike protein. The recombinant virus was thus viable. However, when compared with the original G614 strain, we noticed that the *in vitro* growth kinetics of D614 ISA strain were slower. Such a discrepancy, in different cells lines and at different hours of infection, was already published in a study comparing G614 and D614 strains (Plante *et al*, 2020).

The utilization of tagged viruses for neutralization or antiviral assays has been widely promoted, as the presence of a reporter sequence enables direct monitoring of virus replication (Hou *et al*, 2020b; Xie *et al*, 2020b). Viruses that incorporate a reporter tag can be valuable tools to characterize small molecules or nAbs that may inhibit virus replication, as the virus load can be monitored directly without the need for endpoint quantification of released genetic or infectious material. In this study, an exogenous sequence reporter was inserted in the viral genome, substituting the non‐essential ORF3a by the mCherry‐coding sequence. The rescued virus was viable and fluorescence was detected 48 h pi. As already shown in a previous study (Rihn *et al*, 2021), the presence of the mCherry‐coding sequence in SARS‐CoV‐2 appeared to be stable for at least five passages in VeroE6 cells. Such recombinant virus is thus well‐adapted to improve and facilitate the process of seroneutralization assays or *in vitro* antiviral screening. *In vitro* assays of the mCherry fluorescent strain on a panel of COVID‐19‐positive and ‐negative human sera or against the antiviral drug, Remdesivir, demonstrated identical results with the wild‐type strain. These procedures, therefore, open up new opportunities to implement robust and uncomplicated platforms for high‐throughput and low‐cost sero‐epidemiological studies. In line with the seroneutralization assay, the mCherry virus can also be confidently used for antiviral screening and EC_50_ determination of antiviral compounds.

It is well established that golden Syrian hamsters provide a relevant animal model to study SARS‐CoV‐2 infection, pathogenesis, and transmission (Sia *et al*, 2020). In our study, both clinical and rescued SARS‐CoV‐2 strains replicated efficiently in infected hamsters and induced significant clinical symptoms. Interestingly, the ISA European strain induced lower clinical symptoms and lower viral titers compared with the corresponding clinical strain and the ISA D614 strain. Differences in viral fitness observed *in vivo* between reverse genetics‐based virus and clinical virus have already been observed particularly for several ISA strains (de Fabritus *et al*, 2015). This can be the consequence of an adaptation of the rescued strain to the environment in comparison to a well‐adapted clinical strain, as also observed for the results observed *in vitro* (Domingo *et al*, 2012). This hypothesis is well supported regarding the genotypic changes (majority and minority mutations) of the rescue ISA European strain we observed after two passages in cell culture.

Clinical evaluation to assess the role of the residue—G or D—at position 614 in a hamster model has already been established and showed no difference (Hou *et al*, 2020a; Plante *et al*, 2020) between G614 and D614 virulence. Therefore, the precise role of the amino acid in position 614 of the spike protein on viral fitness in cell culture or *in vivo* remains to be further elucidated. Thus, a comparative evaluation of viral strains between viruses of common origin, for example, recombinant viruses or clinical should be performed.

In conclusion, we report an original and rapid reverse genetic procedure suitable for rescuing infectious coronaviruses under relatively simple operating conditions. In comparison to “conventional” reverse genetics technologies used to produce infectious clones, infectious viruses generated by ISA can be obtained with no need of labored technical steps (i.e., *in vitro* synthesis of full‐length 30 kb RNA transcripts, cloning and sub‐cloning of full length or fragmented genomes in bacteria). Similarly, compared to the closest techniques that require a circular polymerase extension reaction (CPER) step of DNA fragments (Amarilla *et al*, 2021; Torii *et al*, 2021), recombination of the DNA fragments produced after PCR in the ISA method is directly performed *in cellulo*, making it lighter in term of technical steps.

The method was shown to be suitable for the *de novo* rescue of wild‐type viruses and for the generation of mutated or engineered viruses. This unique and simplified reverse genetics method has the potential to accelerate significantly our comprehension of human and animal coronavirus pathogenesis, epidemiology, immunology, and evolution. Moreover, it could also facilitate the further development of therapeutic and vaccine strategies.

## Materials and Methods

### Cells

Baby Hamster Kidney 21 cells (BHK‐21; ATCC CCL‐10) were cultured in Minimal Essential Medium (MEM; Life Technologies) with 5% heat‐inactivated fetal calf serum (FCS), 1% L‐glutamine (200 mM; Life Technologies), 5% Tryptose Phosphate Broth (TPB; Life Technologies), and 1% Penicillin/Streptomycin (P/S; 5,000 U/ml; 5 mg/ml). VeroE6 cells (ATCC CRL‐1586) were grown in MEM supplemented with 5% FCS, 1% l‐glutamine, 1% P/S, and 1% non‐essential amino acids (NEAA; Life Technologies). Feline embryonic fibroblast cells (FeA) were cultured in Dulbecco's Modified Eagle Medium (DMEM; Life Technologies) with 10% FCS and 1% P/S. Feline pulmonary epithelial cells (AK‐D; ATCC CCL‐150) were cultured in a mix of Ham's F12 (F12) and Leibovitz's (L‐15) media (v/v) with 7% FCS and 1% PS. Cells were grown at 37°C in an atmosphere containing 5% CO_2_.

### Viral strains

A clinical SARS‐CoV‐2 European strain was provided by Pr. Christian Drosten (Charité, Berlin) from the European Archive Collection (human isolate BetaCoV/Germany/BavPat1/2020 p.1; reference: 026V‐03883). FeCoV was obtained from the American Type Culture Collection (ATCC reference: VR‐2126). SARS‐CoV‐2 and FeCoV clinical samples were first passaged on VeroE6 and FeA cells, respectively. Culture flasks of confluent VeroE6 and FeA cells were infected with the clinical SARS‐CoV‐2 at a multiplicity of infection (moi) of 0.001) and FeCoV (moi 0.01), respectively. Cells were washed twice (HBSS) 1 h after infection and 4 ml of medium was added. Cell supernatant media were sampled at 48 h pi, clarified by centrifugation, aliquoted, and stored at −80°C. All experiments were approved by the French ‘Ministère de l’Enseignement Supérieur, de la Recherche et de l’Innovation (agreement number 7155). All *in vitro* experiments were conducted in a biosafety level‐3 (BSL3) facility‐compliant laboratory by the faculty of Médecine of Marseille, France.

### Preparation of subgenomic cDNA fragments

Based on full‐length sequences, eight overlapping synthetic DNA fragments were designed and *de novo* synthesized by the manufacturers (Genscript or Thermo Fisher Scientific). The first and last fragments were *de novo* synthesized with the human cytomegalovirus promoter (pCMV) and the hepatitis delta virus ribozyme followed by the simian virus 40 polyadenylation signal (HDR/SV40pA) at their 5′ and 3′ extremities, respectively. To construct the D614 and the red fluorescent reporter strains, synthetic DNA fragments with the following modifications were also *de novo* synthesized: according to a Wuhan SARS‐CoV‐2 genome (Genbank accession number: NC045512), the codon GGT of the Glycine located at position 614 of the spike protein, from Fragment 7, was replaced by the codon GAT for an Aspartic acid and the new fragment (Fragment 7 D614) was *de novo* synthesized (Thermo Fisher Scientific). Finally, downstream of the regulatory sequence of ORF3a from the fragment 7 D614, the coding sequence of the red fluorescent protein (mCherry; Genbank accession number: AY678264) was inserted (position 25,392–26,221 in the genome sequence) and *de novo* synthesis (Thermo Fisher Scientific).

cDNAs were amplified from these *de novo* synthetic viral fragments as templates. A Super Fidelity PCR polymerase kit (Thermo Fisher Scientific) was used. Primer sequences and positions on the genome are described in Appendix Table S3. The final mixture contained 25 µl of reaction mix, 2 µl of DNA (1 ng/µl), 100 nM of each primer, and 20 µl nuclease‐free water. PCR reactions were performed on a Biometra TProfessional Standard Gradient thermocycler with the following conditions: 98°C for 2 min followed by 35 cycles of 98°C for 10 s, 55–60°C (see Appendix Table S4) for 10 s, 72°C for 30s/kb, and 72°C for 5 min, and a preliminary step of 50°C for 10 min for the RT‐PCR. Amplicons were purified (Monarch^®^ PCR & DNA Cleanup Kit; New England Biolabs) and the size of PCR products was verified by gel electrophoresis. All products were sequenced to ensure that the genotypic integrity of each cDNA fragment produced by PCR was accurate before transfection.

### Cell transfection

Equimolar proportions of the eight subgenomic cDNA fragments encompassing the SARS‐CoV‐2 or the FeCoV genomes were pooled (from ~25 to 55 ng of each fragment, depending on fragment length; final quantity of all pooled fragments: 300 ng) and transfected into 96 wells of subconfluent BHK‐21 cells and a coculture of BHK‐21 plus FeA cells, respectively, using Lipofectamine 3000 (Thermo Fischer Scientific). The Lipofectamine and DNA were incubated for 45 min at room temperature and incubated with the cells for a further 24 h at 37°C. These cell lines were selected after optimizing a panel of conditions in parallel (Appendix Table S5) and were thus selected for the rest of the study. For SARS‐CoV‐2, a suspension of VeroE6 cells was added 24 h after transfection and then incubated for 5 days at 37°C in 5% CO_2_. For FeCoV, fresh medium was added 24 h after transfection and then incubated for 5 days at 37°C in 5% CO_2_. Cell supernatant media were harvested and serially passaged twice to ensure the complete disappearance of the DNA used during transfection. Serial passages were performed by inoculating clarified supernatant media onto subconfluent VeroE6 and AK‐D cells for SARS‐CoV‐2 and FeCoV, respectively: after incubation for 1 h, cells were washed twice using Hanks' Balanced Salt solution (HBSS; Gibco), fresh medium was added, and plates were incubated for 2 days. After the last passage, cell supernatant media were harvested, clarified by centrifugation, aliquoted, and stored at −80°C. These virus stocks were used to perform quantification of viral RNA, TCID_50_ assay, sequencing and determination of kinetic reproduction. To ensure that the mCherry reporter gene remained stable during the course of these experiments, three additional passages were performed in VeroE6 cells under the same conditions as described below. The full mCherry coding sequence (= 711 bp) was RT‐PCR amplified under the following conditions: 98°C for 2 min followed by 35 cycles of 98°C for 10 s, for 58°C for 10 s, 72°C for 30 s, and 72°C for 5 min with a preliminary step of 50°C for 10 min for the RT‐PCR. The size of the PCR products was checked by gel electrophoresis and sequenced to ensure the stability of the insertion. Additionally, the coding sequence of the spike protein, nucleocapsid, membrane, and envelope proteins were amplified by RT‐PCR under the following conditions: 98°C for 2 min followed by 35 cycles of 98°C for 10 s, 58°C for 10 s, 72°C for 2 min (for the spike), 1 min (for the nucleocapsid) or 30 s (for the membrane and envelope proteins), and 72°C for 5 min with a preliminary step of 50°C for 10 min for the RT‐PCR. The size of these PCR products was checked by gel electrophoresis. All primer sequences are described in Appendix Table S6.

### Remdesivir antiviral activity on clinical and ISA SARS‐CoV‐2 strains

The antiviral efficacy of Remdesivir on SARS‐CoV‐2 strains was assessed by determining the 50% effective concentration (EC_50_) as described in Touret *et al*, 2020 (Touret *et al*, 2020). Briefly, one day prior to infection, 5 × 10^4^ VeroE6 cells were seeded in 100 μl assay medium (containing 2.5% FCS) in 96‐well plates. The next day, eight 2‐fold serial dilutions of Remdesivir (from 20 to 0.15 µM, in three technical replicates (BLDPHARM, Shanghai, China) were added to the cells (25 μl/well, in assay medium). Four virus control wells were supplemented with 25 μl of assay medium. After 15 min, 25 μl of a calibrated virus mix diluted in medium was added to each well. Four cell control wells (i.e., with no virus) were supplemented with 25 μl of assay medium. Plates were incubated for 2 days at 37°C prior to quantification of the viral genome by real‐time RT‐PCR as described below. For fluorescence experiments, plates were analyzed after removing the supernatant medium, washing twice with PBS, and fixed for 2 h with PFA (4%). Quantification of the fluorescence was performed using the Incucyte^®^ S3 Live‐Cell Analysis Systems (Sartorius) according to the manufacturer’s instructions, with an acquisition time of 800 ms for the red channel. The fluorescence intensities from multiple areas were obtained using the Incucyte 2020B software (Sartorius). Dose–response curves were generated using GraphPad Prism 7.00 with a four‐parameter linear regression. EC_50_ was estimated using logarithmic interpolation also with GraphPad Prism 7.00. This experiment was performed in two technical replicates.

### Human sera

Twenty‐four human sera were tested for the presence of SARS‐CoV‐2‐neutralizing antibodies (nAbs). These samples were surplus to requirements from a SARS‐CoV‐2 research program (Hospital Clinical Research Program 2020‐A01653‐36, funded by the Assistance Publique ‐ Hôpitaux de Marseille and approved by the Ethics Committee of South‐West and Overseas France II) where participants provided written consent from research on SARS‐COV‐2 humoral immunity. All human sera were heat inactivated at 56°C for 30 min before anonymization and testing.

### Cytopathic effect and fluorescent‐based neutralization assay

The level of SARS‐CoV‐2 nAbs was determined based on cpe and fluorescence intensity. One hundred and ten microliters of two‐fold serial‐diluted serum was pre‐incubated with 110 µl of 1,000 TCID_50_/ml of SARS‐CoV‐2 strains in 5% FBS in DMEM for 60 min at 37°C. The virus–serum mixtures were then added to 96‐well plates of confluent monolayer Vero‐E6 cells for 5 days at 37°C with 5% CO_2_. The neutralization titer was defined as the highest dilution that inhibited the production of distinct cpe or fluorescence. Samples with nAbs titers ≤ 10 were considered negative. A negative control was added in which, the sera were pre‐incubated with a non‐fluorescent SARS‐CoV‐2 strain (ISA D614 SARS‐CoV‐2) as described previously and added onto confluent monolayers of Vero‐E6 cells for 5 days at 37°C with 5% CO_2_. We ensured that no fluorescence was observed in this control. Quantification of the fluorescence and dose–response curves were performed as described above.

### *In vivo* experiments with SARS‐CoV‐2

*In vivo* experiments in a hamster model were performed as previously described (Driouich *et al*, 2021). All experiments were approved by the local ethical committee (C2EA—14) and the French ‘Ministère de l’Enseignement Supérieur, de la Recherche et de l’Innovation’ (APAFIS#23975) and performed in accordance with the French national guidelines and the European legislation covering the use of animals for scientific purposes. Each *in vivo experiments* were conducted in a BSL3 at the faculty of Médecine of Marseille, France.

#### Animal handling

Female Syrian hamsters aged 3 weeks were provided by Janvier Labs. Animals were maintained in ISOcage P ‐ Bioexclusion System (Techniplast) with unlimited access to water/food and 14 h/10 h light/dark cycle. Every day, animals were weighed and monitored for the duration of the study to detect the appearance of any clinical signs of illness/suffering. Virus inoculation was performed under general anesthesia (isoflurane). Lungs and blood were collected after euthanasia (cervical dislocation) which was also carried out under general anesthesia (isofluorane).

#### Hamster infection

Groups of four anesthetized animals (4 weeks old) were intranasally infected with 50 µl containing 10^3^ TCID_50_ of virus in 0.9% sodium chloride solution. The mock group was intranasally inoculated with 50 µl of 0.9% sodium chloride solution.

#### Organ collection

Lung and blood samples were collected immediately after the time of sacrifice. Left pulmonary lobes were first washed in 10 ml of 0.9% sodium chloride solution and then transferred to a 2‐ml tube containing 1 ml of 0.9% sodium chloride solution and 3‐mm glass beads. They were crushed using the Tissue Lyser machine (Retsch MM400) for 20 min at 30 cycles/s and then centrifuged for 10 min at 16,200 *g*. Supernatant media were transferred to a 2‐ml tube, centrifuged for 10 min at 16,200 *g*, and stored at −80°C. One milliliter of blood was harvested in a 2‐ml tube containing 100 µl of 0.5 M EDTA (ThermoFischer Scientific). Blood was centrifuged for 10 min at 16,200 *g* and stored at −80°C.

### Viral complete genome sequencing

Sequencing was performed from the cell culture supernatant medium to determine entire viral genome sequences and ensure no drift in the consensus sequence. Extraction of nucleic acid was performed using the EZ1 advanced XL machine (Qiagen) with the EZ1 Virus Mini Kit v2.0 (Qiagen). The first random amplification of the viral genomic nucleic acids was performed as previously described (Aubry *et al*, 2014). Amplification was also performed using specific primers and the RT‐PCR kit. The SuperScript™ IV One‐Step RT‐PCR System kit (ThermoFisher Scientific) was used. Sequences of primers designed from the full‐length genomes are available in Appendix Table S6. PCR products were purified (Monarch^®^ PCR & DNA Cleanup Kit; New England Biolabs) and pooled in equimolar proportions. After Qubit quantification using Qubit^®^ dsDNA HS Assay Kit and Qubit 2.0 fluorometer (ThermoFisher Scientific), amplicons were sonicated to produce fragments of about 200 bp in length. Libraries were built by adding barcodes, for sample identification and primers to fragmented DNA using AB Library Builder System (Thermo Fisher Scientific). To pool the barcoded samples in equimolar proportions a quantification step was performed using real‐time PCR with Ion Library TaqMan™ Quantitation Kit (Thermo Fisher Scientific). Emulsion PCR of the pools and loading on a 530 chip was achieved using the automated Ion Chef instrument (ThermoFisher). Sequencing was performed using the S5 Ion torrent technology v5.12 (Thermo Fisher Scientific) following the manufacturer’s instructions (Rothberg *et al*, 2011). Consensus sequence was obtained after trimming of reads (reads with quality score < 0.99, length < 100 bp were removed and the first and last 30 nucleotides were removed from the reads) and mapping of the reads on a reference (determined following blast of De Novo contigs) using CLC genomics workbench software v.20 (Qiagen). *De novo* contigs were produced to ensure that the consensus sequence was not affected by the reference sequence. Mutations with frequencies > 50% in sequencing reads were defined as majority mutations. Mutations with frequencies between 5 and 50% in sequencing reads were identified and considered as minority mutations.

### RT‐qPCR assays

Viral RNA was isolated from 100 μl of cell supernatant medium using a QIAamp Vira RNA kit and RNase‐Free DNase Set on the automated QIAcube (Qiagen) facility, following the manufacturer’s instructions. Relative quantification of genomic viral RNA was performed using the express One‐Step SuperScript^®^ RT‐qPCR (ThermoFisher Scientific). Additionally, for SARS‐CoV‐2, the subgenomic expression of the E gene was analyzed and quantified following Wölfel *et al* (2020) with the sequence of the leader‐specific, reverse, and probe sequences targeting the E gene described in Appendix Table S7. To isolate viral RNA from tissues, 100 µl of organ clarified homogenates, spiked with 10 µl of internal control (bacteriophage MS2) (Ninove *et al*, 2011), were transferred into an S‐block containing the recommended volumes of VXL, proteinase K, and RNA carrier. The RT‐qPCR reaction mixture (for SARS‐CoV‐2 and MS2 viral genome detection) was processed using the GoTaq Probe 1‐Step RT‐qPCR kit (Promega) and contained 5 μl of Master Mix 2X, 0.25 μl of each primer (500 nM), 0.07 μl of probe (75 nM), 0.2 μl of GoScript RT mix, 0.4 µl of H_2_O, and 3.8 μl of extracted nucleic acids. Assays were performed using the QuantStudio 12K Flex Real‐Time PCR machine (ThermoFisher Scientific) with the following conditions: 50°C for 15 min and 95°C for 2 min, followed by 45 cycles of 95°C for 3 s and 60°C for 30 s. Data collection occurred during the 60°C step. The amount of viral RNA was calculated from standard curves using synthetic RNA. The primers and probes used are described in Appendix Table S7.

### Tissue Culture Infectious Dose 50 (TCID50) assay

Subconfluent cultures of VeroE6 and AK‐D cells in 96‐well culture microplates were used for TCID_50_ determination of SARS‐CoV‐2 and FeCoV, respectively. Cells were inoculated with 100 or 150 μl per well of serial dilutions of each sample (four‐fold or 10‐fold dilutions when analyzing lung clarified homogenates or cell supernatant media, respectively) and incubated for 3–6 days for each virus. Each row included six wells of the dilution and two negative controls. The presence of cpe in each well was used to determine TCID_50_/ml. The determination of the TCID_50_/ml for both viruses was performed using the Reed and Muench method (Reed & Muench, 1938).

### Virus replication kinetics

Infections at input multiplicities of 0.001 and 0.01 were performed using subconfluent VeroE6 or AK‐D cells for SARS‐CoV‐2 and FeCoV, respectively. Cells were washed twice (HBSS) for 4 and 1 h after infection with SARS‐CoV‐2 and FeCoV, respectively, and fresh medium was added. Cell supernatant media were sampled every 12 h for up to 48 h, clarified by centrifugation, aliquoted, and stored at −80°C. They were then analyzed using the RT‐qPCR assay as described above.

### Statistical analyses

The correlation between fluorescence and nAbs titers was determined using a non‐linear regression model in the software Prism 7 (GraphPad). The Pearson correlation coefficient (R^2^) and *P* value were calculated using the default settings in the software Prism 7.00. Statistical analysis and graphical representation were performed using GraphPad Prism 7.00. *P* values ≤ 0.05 were considered statistically significant.

### Ethical statement

Dual Use Research of Concern (DURC) aspects of this study have been carefully considered by our institution. All experiments were approved by a local committee and the French ‘Ministère de l’Enseignement Supérieur, de la Recherche et de l’Innovation’ with appropriate agreements described above (see sections 'Viral strains' and '*In vivo* experiments with SARS‐CoV‐2').

## Disclosure and competing interests statement

The authors declare that they have no conflict of interest.

## Supporting information



Appendix

Expanded View Figures PDF

## Data Availability

This study includes no data deposited in external repositories.
