# Supplementary material for: A simple reverse genetics method to generate recombinant coronaviruses
Source: EMBO Rep. 2022 Mar 3;23(5):e53820. doi: 10.15252/embr.202153820 (PMC9066064; doi:10.15252/embr.202153820)
Supplement: Supplementary file 1 — Appendix [file EMBR-23-e53820-s001.pdf]

Appendix for 'A simple reverse genetics method to generate recombinant coronaviruses'.

Table of contents

Appendix Table S1. Quantification of subgenomic viral RNA (sgRNA) of each clinical and recombinant SARS-CoV-2 by RT-qPCR. The amount of subgenomic viral RNA copies was expressed as log_{10} sgRNA copies/ml. .... **page 2**

Appendix Table S2. Neutralizing antibody (nAb) titers using ISA D614 and mCherry D614 SARS-CoV-2 strains. A total of 24 human sera were two-fold diluted and incubated with the ISA D614 and mCherry D614 strains. nAb titers were recorded at 5 days post-infection. Titers were defined as the highest dilution that inhibited the production of distinct cpe with the ISA D614 SARS-CoV-2 or fluorescence with the fluorescent mCherry D614 SARS-CoV-2. .... **page 3**

Appendix Table S3. Primers used to amplify cDNA from *de novo* synthesized fragments. .... **page 4**

Appendix Table S4. Melting temperature (Tm) of primers during synthetic DNA fragments amplification. .... **page 6**

Appendix Table S5. Cell lines for which transfections had been attempted to rescue SARS-CoV-2 and FeCoV. Transfections were performed either on one cell line, (condition 1) two cell lines (condition 2) or one cell line with addition of a second cell line 24 hours after transfection (condition 3). .... **page 7**

Appendix Table S6. Primers used for RT-PCR to amplify RNA from viral supernatant. .... page **8**

Appendix Table S7. Primers and probes used for real time RT-qPCR. gRNA: genomic RNA; sgm: subgenomic RNA .... **page 9**

Appendix Table S8. Individual data from in vivo experiments. Animal weights, lung infectious titers, lung viral RNA yields and plasmatic viral RNA yields. .... **page 10**

Appendix Table S9. Statistical analysis from in vivo experiments .... **page 11**

Appendix Table S1. Quantification of subgenomic viral RNA (sgRNA) of each clinical and recombinant SARS-CoV-2 by RT-qPCR**.** The amount of subgenomic viral RNA copies was expressed as $\log_{10}$ sgRNA copies/ml.

		**Amount of sgRNA**
**SARS-CoV-2**	Clinical European	7.8 +/- 0.2
	ISA European	7.6 +/- 0.1
	ISA D614	8.0 +/- 0.4
	mCherry ISA D614	6.8 +/- 0.2

Appendix Table S2. Neutralizing antibody (nAb) titers using ISA D614 and mCherry D614 SARS-CoV-2 strains. A total of 24 human sera were two-fold diluted and incubated with the ISA D614 and mCherry D614 strains. nAb titers were recorded at 5 days post-infection. Titers were defined as the highest dilution that inhibited the production of distinct cpe with the ISA D614 SARS-CoV-2 or fluorescence with the fluorescent mCherry D614 SARS-CoV-2.

	nAb titers	
**Sera n°**	**ISA D614 strain**	**mCherry D614 strain**
**1**	40	20
**2**	40	40
**3**	40	40
**4**	40	40
**5**	40	80
**6**	40	80
**7**	40	80
**8**	40	80
**9**	80	80
**10**	80	80
**11**	80	80
**12**	80	80
**13**	80	80
**14**	80	80
**15**	80	80
**16**	160	160
**17**	160	160
**18**	160	160
**19**	≤10	≤10
**20**	≤10	≤10
**21**	≤10	≤10
**22**	≤10	≤10
**23**	≤10	≤10
**24**	≤10	≤10

Appendix Table S3. Primers used to amplify cDNA from *de novo* synthesized fragments.

Virus	Fragment	Primer Forward	Primer Reverse	Position	Length (bp)	Overlap (bp)
**SARS-CoV-2**	1	TCAATATTGGCCATTAGCCATA	CACATATGTATTGTTCTTTTTACCC	start-3042	3777	97
	2	GCATTGATTTAGATGAGTGGAGTATG	CATCTTTTAAATGGGATTTAACTGC	2946-7224	4278	82
	3	GTGGTTTAGATTCTTTAGACACC	GGTGCTAGGAGAGTGTGGACAC	7143-11396	4253	87
	4	GTGTTATGTATGCATCAGCTG	ATTGAAATCAATAGCCGCCAC	11310-15185	3875	56
	5	AGTACTATGACCAATAGACAGTTTC	CCACACACCAGCTTTTGATAAAAGT	15130-19341	4211	88
	6	GCTATCTAACCTTAACTTGCC	GAACTTCTACATGCACCAGC	19254-23127	3873	136
	7	GCACACCTTGTAATGGTGTTG	GGATTTGTCTTCTACAATTTGC	22992-26635	3643	110
	8	GCAGATTCCAACGGTACTATTACC	GAAAAAAATGCTTTATTTGTGAAATT	26526-end	3555	
**FeCoV**	1	GAATAAGGGCGACACGGAAA	CAAAAGCGATTCTGATGC	start-3291	4075	129
	2	GGGTGTAGAACTTGAAGGCT	TTCCTACCATGCAACAACCC	3166-6553	3387	115
	3	GCTGGTTTTTGCATGTTGTC	TGCTATGATTCTTAGTTT	6438-10755	4317	100
	4	GTGTTTACCAATTTACGGTT	TGAAGAGTTTTATGCTAGCC	10656-15139	4483	111
	5	GGGTTAAACATCTACAGAAA	GGTAAACCATTATTATTT	15029-20756	5727	88

6	GTATTTTAATAATATAC	GAATAAAAACCAACAGAT	20669-23632	2963	71
7	GCAGTAGCAGTACAGGCTAG	GGTCGGAAGAATAATGAT	23562-27105	3543	91
8	AAATGGCCACACAGGGAC	GCGCTGGCATTGACCCTGAG	27012-end	2573	

Appendix Table S4. Melting temperature (Tm) of primers during synthetic DNA fragments amplification.

Virus	Fragment	Tm (°C)
**SARS-CoV-2**	1	55
	2	55
	3	55
	4	61
	5	60
	6	57
	7	60
	8	60
**FeCoV**	1	60
	2	58
	3	56
	4	58
	5	55
	6	55
	7	58
	8	58

Appendix Table S5. Cell lines for which transfections had been attempted to rescue SARS-CoV-2 and FeCoV. Transfections were performed either on one cell line, (condition 1) two cell lines (condition 2) or one cell line with addition of a second cell line 24 hours after transfection (condition 3).

**Virus**	Condition 1	Condition 2		Condition 3	
	Cell line	Cell line 1	Cell line 2	Cell line 1	Cell line 2
**SARS-CoV-2**	BHK-21	BHK-21	VeroE6	BHK-21	VeroE6
		BHK-21	BGM		
		BHK-21	A549		
		BHK-21	Huh7		
	HEK-293	HEK-293	VeroE6	HEK-293	VeroE6
		HEK-293	BGM		
		HEK-293	A549		
		HEK-293	Huh7		
	BGM	BGM	A549		
			Huh7		
	A549	A549	Huh7		
	Huh7				
**FeCoV**	BHK-21	BHK-21	FeA		
		BHK-21	Fcw		
		BHK-21	A-72		
		BHK-21	FeA		
	HEK-293	HEK-293	FeA		
		HEK-293	AK-D		
		HEK-293	Fcw		
		HEK-293	A-72		
		HEK-293	FeA		
	SW-13	SW-13	FeA		
		SW-13	AK-D		
		SW-13	Fcw		
		SW-13	A-72		
		SW-13	FeA		
	FeA				
	Fcw				
	A-72				

Appendix Table S6. Primers used for RT-PCR to amplify RNA from viral supernatant.

	Primer Forward	Primer Reverse
**SARS-CoV-2**	ATTAAAGGTTTATACCTTCCCAGG	CACATATGTATTGTTCTTTTTACCC
	GCATTGATTTAGATGAGTGGAGTATG	CATCTTTTAAATGGGATTTAACTGC
	GTGGTTTAGATTCTTTAGACACC	GGTGCTAGGAGAGTGTGGACAC
	GTGTTATGTATGCATCAGCTG	ATTGAAATCAATAGCCGCCAC
	AGTACTATGACCAATAGACAGTTTC	CCACACACCAGCTTTTGATAAAAGT
	GCTATCTAACCTTAACTTGCC	GAACTTCTACATGCACCAGC
	GCACACCTTGTAATGGTGTTG	GGATTTGTCTTCTACAATTTGC
	GCAGATTCCAACGGTACTATTACC	ATTTTAATAGCTTCTTAGGAGAATGAC
**mCherry**	ATGGTGAGCAAGGGCGAGG	TGGACGAGCTGTACAAGTAG
**Spike**	CTTGTTAACAACTAAACGAAC	ACGAACTTATGGATTTGTTTATGA
**Nucleocapsid**	GATTTCATCTAAACGAACAAACTAAA	ACTCATGCAGACCACACAAG
**Membrane**	ATGGCAGATTCCAACGGTAC	CAATATTGCTTTGCTTGTACAGTAA
**Enveloppe**	ATGTACTCATTCGTTTCGGAAG	GTTCCTGATCTTCTGGTCTAA
**FeCoV**	ACTTTTAAAGTAAAGTGAGTGTAGCG	GGTGATTCAGGGGATGCAGA
	GGGTGATTCAGGGGATGCAG	AAGCCTGGTTCTTCTGGTGT
	CGTTAATAGTGAGTTGCTTGAGG	GTGTGACGACCCTGACGC
	TTGATGGGCATTGGTGGAGG	ATCTTCCCTACCCAGACCCG
	GTGATGGTACTACAGCTTACGC	CATGCTTTCCACACACCAGC
	GGTAATCCCAAAGGCATCCG	ACTGGTGTTAGTGGAGCTTTT
	TGAAATCCCGTTCGGCATAAC	TGACACTTGTAGGGCCTATGC
	CCATGCGAAAATGATTGGTGGA	TTTCCTTTTGATAGTGATACAC

Appendix Table S7. Primers and probes used for real time RT-qPCR. gRNA: genomic RNA; sgm: subgenomic RNA

Virus	Length (bp)	Primer Forward	Probe	Primer Reverse
**SARS-CoV-2 gRNA**	61	GGCCGCAAATTGCACAAT	CCCCCAGCGCTTCAGCGTTCT	GGAATGTCGCGCATTGG
**SARS-CoV-2 sgm RNA**		CGATCTCTTGTAGATCTGTTCTC	ACACTAGCCATCCTTACTGCGCTTCG	TGTGTGCGTACTGCTGCAATAT
**FeCoV gRNA**	118	GCATGGCTTGCTACGCTCAT	CGCCACCAACGGT	AACACTGCAGTCCGGATTGAG
**Bacteriophage MS2**	100	CTCTGAGAGCGGCTCTATTGGT	CAGACACGCGGTCCGCTATAACGA	GAATTTAGGCTCGTTGTAGGGAAC

Appendix Table S8. Individual data from in vivo experiments. Animal weights, lung infectious titers, lung viral RNA yields and plasmatic viral RNA yields.

Inoculum ($TCID_{50}$)	Strain	Animal ID	Animal weights (g)								Day of	Lung			Plasmatic viral RNA yields
													Infectious titers	Viral RNA yields	
			Day 0	Day 1	Day 2	Day 3	Day 4	Day 5	Day 6	Day 7		Weights (g)	$log10 \frac{Lung\ TCID_{50}/mL}{Lung\ weight}$	$log10 \frac{Lung\ RdRp\ copies}{Lung\ weight}$	$log_{10}\ (Plasmatic\ viral\ RNA\ copies/mL)$ ¤ : Detection threshold at 100 cp/ml
	**Mock**	**568**	66.4	70.2	72.2	73.8	76.6	80.1	81.8	83.4	7	-	-	-	-
	**Mock**	**569**	59.8	63.1	64.8	66.4	67.4	72.9	73,0	74.7	7	-	-	-	-
	**Mock**	**570**	62.1	65.3	68.8	72,0	72.4	75.6	79.2	80.4	7	-	-	-	-
	**Mock**	**571**	63.1	65.5	67,0	71.5	73.5	75.3	77.6	82.5	7	-	-	-	-
**10^3**	**ISA D614**	**3**	57,0	59,0	58.5	59.7	-	-	-	-	3	0.123	6.702	9.656	3.991
**10^3**	**ISA D614**	**4**	63.6	56.4	64.4	59.2	-	-	-	-	3	0.160	6.186	9.335	4.847
**10^3**	**ISA D614**	**5**	56.9	59.1	58,0	60.6	-	-	-	-	3	0.122	6.505	9.293	4.611
**10^3**	**ISA D614**	**6**	58,0	60.1	61,0	61.6	-	-	-	-	3	0.150	6.816	9.546	3.401
**10^3**	**ISA D614**	**7**	56.7	59.1	58.9	61.7	56.8	52,0	49.6	55.9	7	-	-	-	-
**10^3**	**ISA D614**	**8**	56.5	58.5	58.3	59.6	54.8	50.4	49.9	55.6	7	-	-	-	-
**10^3**	**ISA D614**	**9**	56,0	58.3	56.8	58.3	53.6	49.9	51.6	55.3	7	-	-	-	-
**10^3**	**ISA D614**	**10**	61.7	63.4	61.9	63.8	58.2	55,0	57,0	61.8	7	-	-	-	-
**10^3**	**ISA European**	**11**	58.4	60,0	59.1	59.9	-	-	-	-	3	0.162	5.428	8.916	4.770
**10^3**	**ISA European**	**12**	49.3	49.7	48.8	49.8	-	-	-	-	3	0.121	5.555	8.810	5.130
**10^3**	**ISA European**	**13**	55,0	57.3	57,0	59.5	-	-	-	-	3	0.153	6.005	9.089	4.348
**10^3**	**ISA European**	**14**	53.3	55.6	53.9	56.1	-	-	-	-	3	0.131	6.073	9.189	5.755
**10^3**	**ISA European**	**15**	61.4	63.5	61.8	64.5	59.6	58.9	63.3	66.4	7	-	-	-	-
**10^3**	**ISA European**	**16**	53.6	55.5	55.1	56.4	51.9	51.6	55.4	59.7	7	-	-	-	-
**10^3**	**ISA European**	**17**	55,0	57.5	58.5	61.1	60,0	56.4	56.2	61.3	7	-	-	-	-
**10^3**	**ISA European**	**18**	59.3	62,0	60.4	61.7	60.3	56.9	59.1	63.7	7	-	-	-	-
**10^3**	**Clinical European**	**19**	68.1	71.2	70.8	73.2	-	-	-	-	3	0.163	6.780	9.132	4.911
**10^3**	**Clinical European**	**20**	61.1	63,0	61.3	63.9	-	-	-	-	3	0.149	6.418	9.323	4.220
**10^3**	**Clinical European**	**21**	59.2	60.4	59.4	62.1	-	-	-	-	3	0.138	6.552	9.098	¤ 2.000
**10^3**	**Clinical European**	**22**	59.6	63.1	63.1	64.1	-	-	-	-	3	0.147	6.825	9.037	2.384
**10^3**	**Clinical European**	**23**	59.5	62,0	61.1	60.4	56.4	53.4	52.9	59,0	7	-	-	-	-
**10^3**	**Clinical European**	**24**	60.9	62.5	62.5	62.9	60.8	56.9	57.1	61.7	7	-	-	-	-
**10^3**	**Clinical European**	**25**	59.2	62.8	61.8	62.7	62.9	56.5	59.1	64.7	7	-	-	-	-
**10^3**	**Clinical European**	**26**	57.7	60.9	59.5	60.4	57.4	53,0	54.3	57.5	7	-	-	-	-

Appendix Table S9. Statistical analysis from in vivo experiments

	Two way ANOVA for animal weights comparaison	
	Tukey's multiple comparisons test	
Day Post Infection	**Groups**	**Adjusted P value**
**0**	Mock vs. ISA D614 strain	>0,9999
	Mock vs. Clinical European strain	>0,9999
	Mock vs. ISA European strain	>0,9999
	ISA D614 strain vs. Clinical European strain	>0,9999
	ISA D614 strain vs. ISA European strain	>0,9999
	Clinical European strain vs. ISA European strain	>0,9999
**1**	Mock vs. ISA D614 strain	0,8573
	Mock vs. Clinical European strain	0,9946
	Mock vs. ISA European strain	0,935
	ISA D614 strain vs. Clinical European strain	0,9477
	ISA D614 strain vs. ISA European strain	0,997
	Clinical European strain vs. ISA European strain	0,9862
**2**	Mock vs. ISA D614 strain	**0,0028**
	Mock vs. Clinical European strain	**0,0164**
	Mock vs. ISA European strain	**0,0101**
	ISA D614 strain vs. Clinical European strain	0,9402
	ISA D614 strain vs. ISA European strain	0,9772
	Clinical European strain vs. ISA European strain	0,9984
**3**	Mock vs. ISA D614 strain	**0,0003**
	Mock vs. Clinical European strain	**<0,0001**
	Mock vs. ISA European strain	**0,0019**
	ISA D614 strain vs. Clinical European strain	0,7959
	ISA D614 strain vs. ISA European strain	0,9559
	Clinical European strain vs. ISA European strain	0,4842
**4**	Mock vs. ISA D614 strain	**<0,0001**
	Mock vs. Clinical European strain	**<0,0001**
	Mock vs. ISA European strain	**<0,0001**
	ISA D614 strain vs. Clinical European strain	0,2456
	ISA D614 strain vs. ISA European strain	0,0677
	Clinical European strain vs. ISA European strain	0,9268

Day Post Infection	Groups	Adjusted P value
**5**	Mock vs. ISA D614 strain	**<0,0001**
	Mock vs. Clinical European strain	**<0,0001**
	Mock vs. ISA European strain	**<0,0001**
	ISA D614 strain vs. Clinical European strain	0,376
	ISA D614 strain vs. ISA European strain	**0,0001**
	Clinical European strain vs. ISA European strain	**0,0246**
**6**	Mock vs. ISA D614 strain	**<0,0001**
	Mock vs. Clinical European strain	**<0,0001**
	Mock vs. ISA European strain	**<0,0001**
	ISA D614 strain vs. Clinical European strain	0,1
	ISA D614 strain vs. ISA European strain	**<0,0001**
	Clinical European strain vs. ISA European strain	**0,0001**
**7**	Mock vs. ISA D614 strain	**<0,0001**
	Mock vs. Clinical European strain	**<0,0001**
	Mock vs. ISA European strain	**<0,0001**
	ISA D614 strain vs. Clinical European strain	0,2237
	ISA D614 strain vs. ISA European strain	**<0,0001**
	Clinical European strain vs. ISA European strain	**0,0004**

ANOVA table	**SS**	**DF**	**MS**	**F (DFn, DFd)**	**P value**
Interaction	1879	14	134,2	F (14, 63) = 14,9	P<0,0001
Time	1993	7	284,7	F (7, 63) = 31,6	P<0,0001
Column Factor	3219	2	1609	F (2, 9) = 28,52	P=0,0001
Subjects (matching)	507,9	9	56,43	F (9, 63) = 6,264	P<0,0001
Residual	567,6	63	9,009		

	Lung infectious titers			Lung viral RNA Yields			Plasma viral RNA yields		
	**Clinical European strain**	**ISA D614 strain**	**ISA European strain**	**Clinical European strain**	**ISA D614 strain**	**ISA European strain**	**Clinical European strain**	**ISA D614 strain**	**ISA European strain**
**Number of values**	4	4	4	4	4	4	4	4	4
**W**	0,9102	0,9503	0,8652	0,8948	0,9065	0,9641	0,8944	0,9481	0,9901
**P value**	0,4835	0,7182	0,2793	0,4055	0,4638	0,8045	0,4036	0,7046	0,9582
**Passed normality test (alpha=0.05)?**	**Yes**	**Yes**	**Yes**	**Yes**	**Yes**	**Yes**	**Yes**	**Yes**	**Yes**
**P value summary**	ns	ns	ns	ns	ns	ns	ns	ns	ns

Statistical comparaison

	Compared groups	P value	Two-sided statistical test	t-values and Degree of freedom
**Lung infectious titers**	Clinical European strain (n=4) vs ISA D614 strain (n=4)	0,6064	Unpaired t-test	t=0,5435 df=6
	Clinical European strain (n=4) vs ISA European strain (n=4)	**0,0034**	Unpaired t-test	t=4,692 df=6
	ISA European strain (n=4) vs ISA D614 strain (n=4)	**0,0099**	Unpaired t-test	t=3,718 df=6
**Lung viral RNA yields**	Clinical European strain (n=4) vs ISA D614 strain (n=4)	**0,0265**	Unpaired t-test	t=2,924 df=6
	Clinical European strain (n=4) vs ISA European strain (n=4)	0,212	Unpaired t-test	t=1,396 df=6
	ISA European strain (n=4) vs ISA D614 strain (n=4)	**0,0093**	Unpaired t-test	t=3,773 df=6
**Plasma viral RNA yields**	Clinical European strain (n=4) vs ISA D614 strain (n=4)	0,3236	Unpaired t-test	t=1,075 df=6
	Clinical European strain (n=4) vs ISA European strain (n=4)	0,078	Unpaired t-test	t=2,122 df=6
	ISA European strain (n=4) vs ISA D614 strain (n=4)	0,1241	Unpaired t-test	t=1,787 df=6
