# Supplementary material for: A simple reverse genetics method to generate recombinant coronaviruses
Source: EMBO Rep. 2022 Mar 3;23(5):e53820. doi: 10.15252/embr.202153820 (PMC9066064; doi:10.15252/embr.202153820)
Supplement: Supplementary file 2 — Expanded View Figures PDF [file EMBR-23-e53820-s003.pdf]

Expanded View Figures

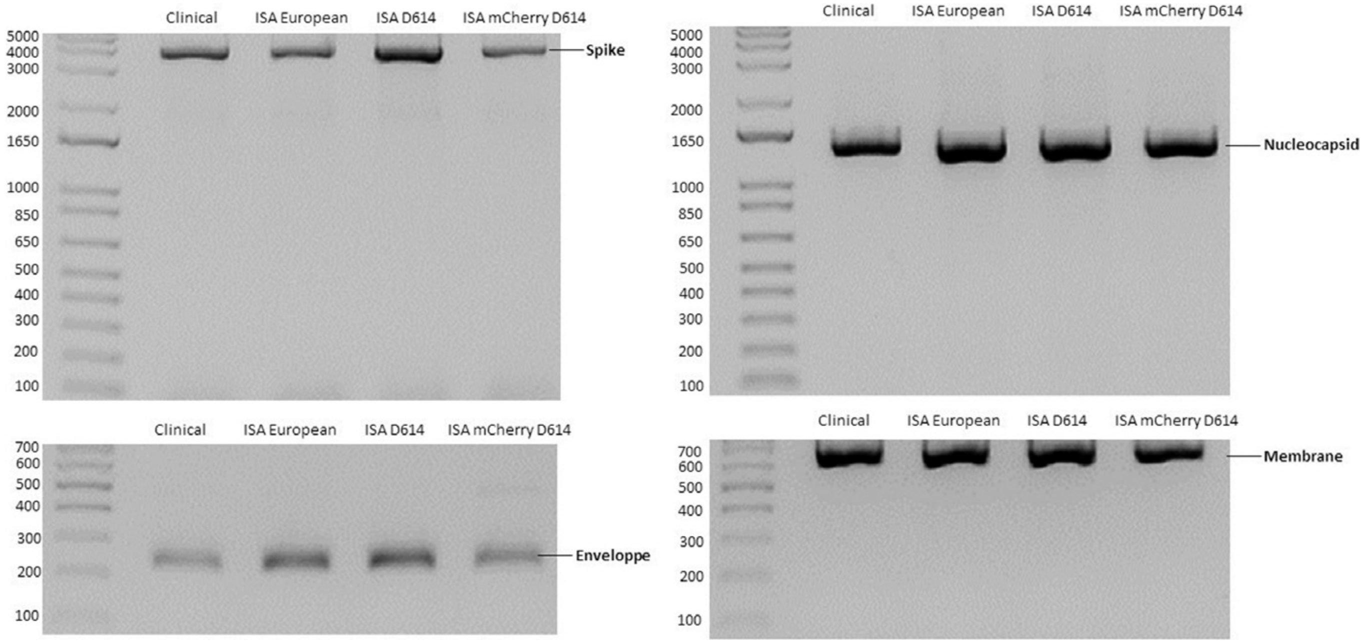

Figure EV1. The spike, nucleocapsid, membrane, and envelope proteins of clinical and recombinant SARS-CoV-2 after two passages in VeroE6 were amplified by RT-PCR from clarified supernatant medium.

© 2022 Unité des Virus Emergents

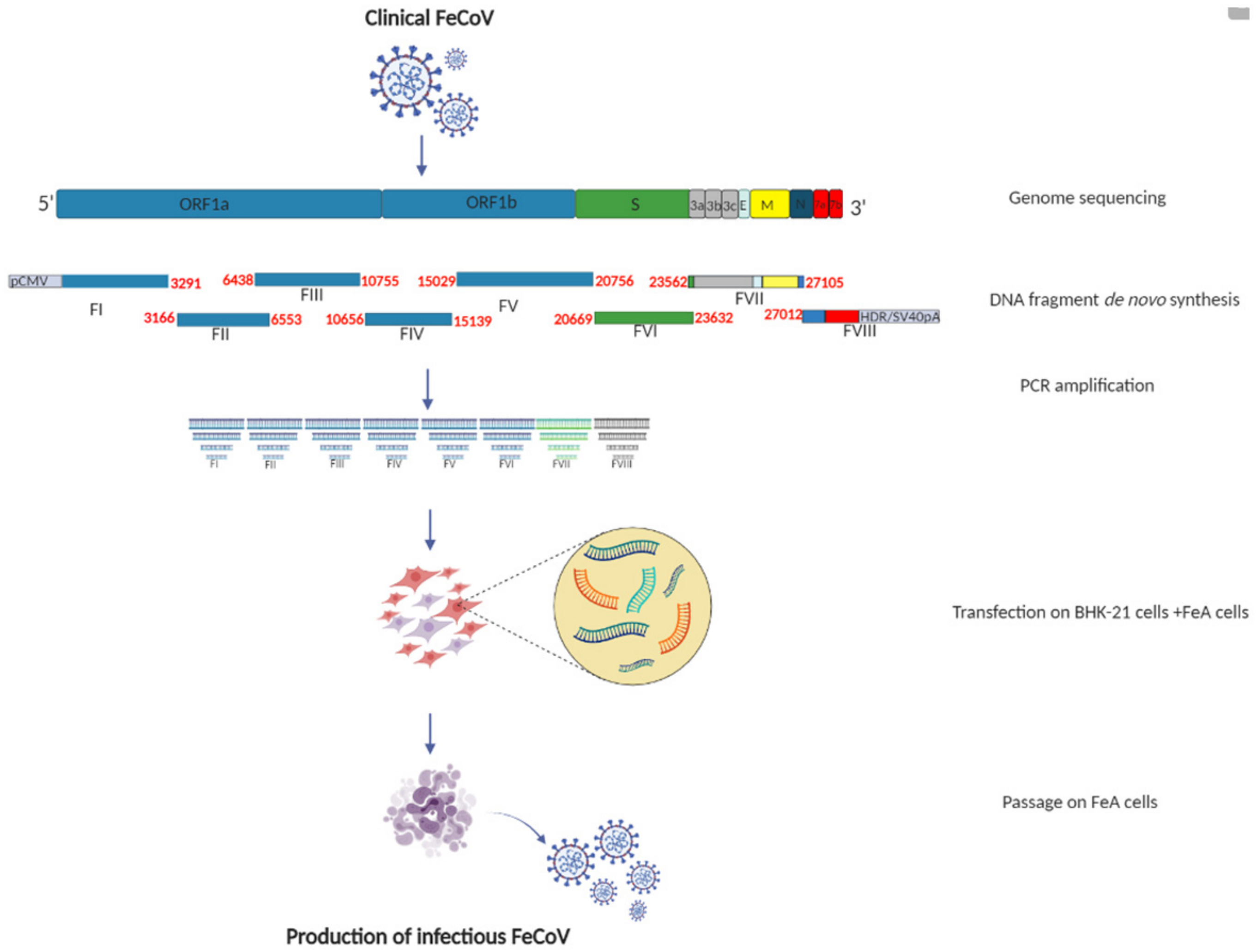

Figure EV2. The ISA method to rescue FeCoV.

FeCoV complete genome sequence was used to design eight overlapping subgenomic viral fragments covering the full genome. Positions on the genome (in nucleotide) are indicated in bold red. This figure was created with BioRender.com.

© 2022 Unité des Virus Emergents
