# Supplementary material for: A simple reverse genetics method to generate recombinant coronaviruses
Source: EMBO Rep. 2022 Mar 3;23(5):e53820. doi: 10.15252/embr.202153820 (PMC9066064; doi:10.15252/embr.202153820)
Supplement: Supplementary file 3 [file EMBR-23-e53820-s002.pdf]

EMBO reports

A simple reverse genetics method to generate recombinant coronaviruses

Julien Mélade, Géraldine Piorkowski, Franck Touret, Toscane Fourié, Jean-Sélim Driouich, Maxime Cochin, Hawa Sophia Bouzidi, Bruno Coutard, Antoine Nougairede, and Xavier de Lamballerie
DOI: 10.15252/embr.202153820

Corresponding author(s): Julien Mélade (julien.melade@univ-amu.fr)

Review Timeline:

Submission Date:	17th Aug 21
Editorial Decision:	17th Sep 21
Revision Received:	3rd Dec 21
Editorial Decision:	10th Jan 22
Revision Received:	2nd Feb 22
Accepted:	8th Feb 22

Editor: Achim Breiling

Transaction Report:

(Note: With the exception of the correction of typographical or spelling errors that could be a source of ambiguity, letters and reports are not edited. Depending on transfer agreements, referee reports obtained elsewhere may or may not be included in this compilation. Referee reports are anonymous unless the Referee chooses to sign their reports.)

Dear Dr. Mélade,

Thank you for the submission of your research manuscript to EMBO reports. We have now received the reports from the three referees that were asked to evaluate your study, which can be found at the end of this email.

As you will see, the referees think that these findings are of interest. However, they have several comments, concerns and suggestions, indicating that a major revision of the manuscript is necessary to allow publication of the study in EMBO reports. As the reports are below, and all their points need to be addressed, I will not detail them here.

Given the constructive referee comments, we would like to invite you to revise your manuscript with the understanding that all referee concerns must be addressed in the revised manuscript or in the detailed point-by-point response. Acceptance of your manuscript will depend on a positive outcome of a second round of review. It is EMBO reports policy to allow a single round of revision only and acceptance of the manuscript will therefore depend on the completeness of your responses included in the next, final version of the manuscript.

Revised manuscripts should be submitted within three months of a request for revision. Please contact me to discuss the revision should you need additional time.

When submitting your revised manuscript, please also carefully review the instructions that follow below.

PLEASE NOTE THAT upon resubmission revised manuscripts are subjected to an initial quality control prior to exposition to re-review. Upon failure in the initial quality control, the manuscripts are sent back to the authors, which may lead to delays. Frequent reasons for such a failure are the lack of the data availability section (please see below) and the presence of statistics based on n=2 (the authors are then asked to present scatter plots or provide more data points).

When submitting your revised manuscript, we will require:

1) a .docx formatted version of the final manuscript text (including legends for main figures, EV figures and tables), but without the figures included. Please make sure that changes are highlighted to be clearly visible. Figure legends should be compiled at the end of the manuscript text.

2) individual production quality figure files as .eps, .tif, .jpg (one file per figure), of main figures and EV figures. Please upload these as separate, individual files upon re-submission.

The Expanded View format, which will be displayed in the main HTML of the paper in a collapsible format, has replaced the Supplementary information. You can submit up to 5 images as Expanded View. Please follow the nomenclature Figure EV1, Figure EV2 etc. The figure legend for these should be included in the main manuscript document file in a section called Expanded View Figure Legends after the main Figure Legends section. Additional Supplementary material should be supplied as a single pdf file labeled Appendix. The Appendix should have page numbers and needs to include a table of content on the first page (with page numbers) and legends for all content. Please follow the nomenclature Appendix Figure Sx, Appendix Table Sx etc. throughout the text, and also label the figures and tables according to this nomenclature.

For more details, please refer to our guide to authors:
http://www.embopress.org/page/journal/14693178/authorguide#manuscriptpreparation

See also our guide for figure preparation:
http://wol-prod-cdn.literatumonline.com/pb-assets/embo-site/EMBOPress_Figure_Guidelines_061115-1561436025777.pdf

3) a .docx formatted letter INCLUDING the reviewers' reports and your detailed point-by-point responses to their comments. As part of the EMBO Press transparent editorial process, the point-by-point response is part of the Review Process File (RPF), which will be published alongside your paper.

4) a complete author checklist, which you can download from our author guidelines (https://www.embopress.org/page/journal/14693178/authorguide). Please insert page numbers in the checklist to indicate where the requested information can be found in the manuscript. The completed author checklist will also be part of the RPF.

Please also follow our guidelines for the use of living organisms, and the respective reporting guidelines:
http://www.embopress.org/page/journal/14693178/authorguide#livingorganisms

5) that primary datasets produced in this study (e.g. RNA-seq, ChIP-seq, structural and array data) are deposited in an appropriate public database. If no primary datasets have been deposited, please also state this a dedicated section (e.g. 'No primary datasets have been generated and deposited'), see below.

See also: http://embor.embopress.org/authorguide#datadeposition

Please remember to provide a reviewer password if the datasets are not yet public.

The accession numbers and database should be listed in a formal "Data Availability" section (DAS - placed after Materials & Methods) that follows the model below. This is now mandatory (like the COI statement). Please note that the Data Availability Section is restricted to new primary data that are part of this study.

Data availability

The datasets produced in this study are available in the following databases:

- RNA-Seq data: Gene Expression Omnibus GSE46843 (https://www.ncbi.nlm.nih.gov/geo/query/acc.cgi?acc=GSE46843)
- [data type]: [name of the resource] [accession number/identifier/doi] ([URL or identifiers.org/DATABASE:ACCESSION])

*** Note - All links should resolve to a page where the data can be accessed. ***

Moreover, I have these editorial requests:

6) We strongly encourage the publication of original source data with the aim of making primary data more accessible and transparent to the reader. The source data will be published in a separate source data file online along with the accepted manuscript and will be linked to the relevant figure. If you would like to use this opportunity, please submit the source data (for example scans of entire gels or blots, data points of graphs in an excel sheet, additional images, etc.) of your key experiments together with the revised manuscript. If you want to provide source data, please include size markers for scans of entire gels, label the scans with figure and panel number, and send one PDF file per figure.

7) Our journal encourages inclusion of *data citations in the reference list* to directly cite datasets that were re-used and obtained from public databases. Data citations in the article text are distinct from normal bibliographical citations and should directly link to the database records from which the data can be accessed. In the main text, data citations are formatted as follows: "Data ref: Smith et al, 2001" or "Data ref: NCBI Sequence Read Archive PRJNA342805, 2017". In the Reference list, data citations must be labeled with "[DATASET]". A data reference must provide the database name, accession number/identifiers and a resolvable link to the landing page from which the data can be accessed at the end of the reference. Further instructions are available at: http://www.embopress.org/page/journal/14693178/authorguide#referencesformat

8) Regarding data quantification and statistics, can you please specify, where applicable, the number "n" for how many independent experiments (biological replicates) were performed, the bars and error bars (e.g. SEM, SD) and the test used to calculate p-values in the respective figure legends. Please provide statistical testing where applicable, and also add a paragraph detailing this to the methods section. See:
http://www.embopress.org/page/journal/14693178/authorguide#statisticalanalysis

9) Please also note our reference format:
http://www.embopress.org/page/journal/14693178/authorguide#referencesformat

10) For microscopic images, please add scale bars of similar style and thickness to all the microscopic images, using clearly visible black or white bars (depending on the background). Please place these in the lower right corner of the images. Please do not write on or near the bars in the image but define the size in the respective figure legend.

11) Please add up to five keywords to the title page.

12) Please add a section to the manuscript text indicating the author contribution. Please order the manuscript sections like this: Title page - Abstract - Introduction - Results - Discussion - Materials and Methods - DAS - Acknowledgements - Author contributions - Conflict of interest - References - Figure legends - Expanded View Figure legends

13) Please include the funding information into the Acknowledgements and make sure that all the funding information is also entered into the online submission system and is complete and similar to the one in the manuscript text file.

14) Finally, please add clear statements to the methods section indicating that all safety standards have been followed, i.e. that also the animal work was conducted in a BSL3 laboratory, and that the approval of the animal experiments included a permission for the specific use of SARS-CoV-2.

I look forward to seeing a revised version of your manuscript when it is ready. Please let me know if you have questions or comments regarding the revision.

Yours sincerely

Achim Breiling
Editor
EMBO Reports

Referee #1:

In their manuscript "A simple reverse genetics method to generate recombinant coronaviruses", Mélade et al. describe a method called Infectious Subgenomic Amplicons (ISA), used to rescue two coronaviruses, SARS-CoV-2 and the feline enteric coronavirus. This method had been described before and used by the same group to generate infectious viral clones for several other RNA viruses (e.g.: flavivirus, picornavirus, alphavirus). Although not novel, this method appears to be much simpler and faster to generate recombinant clones of SARS-CoV-2 than all other methods described so far (Thi Nhu Tao et al., 2020, Hou et al., 2020, Rhin et al., 2021 and Xie et al. 2020).

The main findings presented in this article are that 1) the ISA method provides a fast and easy way to rescue SARS-CoV-2 and generate new infectious clones of coronaviruses that have been known to be difficult to rescue and that 2) the infectious SARS-CoV-2 clones produced by ISA are reliable tools to study SARS-CoV-2 biology. These findings are potentially of great interest to the scientific community, in particular to molecular virologists.

However, in its current version, the article does not provide enough information allowing a fair assessment of the work. First, the description of the ISA method itself is unclear. Second, the assessment of the viral clones' genetic stability is limited (cut-off at 50% after 2 passages). Third, the assays used to test the infectious clones generated by ISA are also unclear (see below the Main Comments section). Finally, the authors do not explain how their statistical tests are suited for their experiments (two way ANOVA, Sidak's test correction, "Statistical analysis W"...) which contributes to the difficulty to assess their conclusions.
The manuscript would greatly benefit from improvements and clarification on all these issues.

Main comments:

Genetic stability:
The authors evaluate the genetic stability of their infectious clones generated by ISA, by performing full genome viral sequencing (Ion Torrent) and report only 2 non-synonymous mutations. This is better than what has been reported for other SARS-CoV-2 rescue platforms (Thi Nhu Tao et al., 2020, Silvas et al 2021). However, the authors set their cut off for mutations at 50%. Viral RNA genetic landscapes can be very diverse and mutations under 50% that can easily hide important viral genetic heterogeneity. Since the authors only performed the sequencing experiment once, it is important that they provide the sequencing results and more importantly report all mutations location and nature (synonymous and non-synonymous), represented in at least 10% of the population.

Neutralizing antibody and antiviral assays:
The method for the fluorescent based neutralization assay is not clear. Also the authors mention "dose-response curves generated using GraphPad Prism 7.00 with a four parameters linear regression model." but they are not shown and their purpose is unclear. The author should describe their negative control for fluorescent, the exact number of areas scanned per well and for each condition and what was considered as an absence of fluorescence for neutralization assay. Similarly, what is the reference signal for the percentage of fluorescence signal in the y axis in Figure 4b?
Figure 5: the legend says that the "EC50 values presented are from three and two independent experiments respectively". However, the figure shows 5 data points for each series at each concentration.
ISA method:
In their method section, the authors describe using "A equimolar mix (300ng) of subgenomic cDNA fragments of SARS-CoV-2" (line 132). It is unclear if they mean a total of 300ng cDNA for the combined 8 amplicons reconstituting the viral genome or that 300ng of each amplicon was added to the transfection mix per well. In line 430, they say that the ISA experiment is performed using a "a total of ~30 µg of mixed PCR fragments" in a 96 well plate. Does it mean that the ISA is actually performed using a total of 30ug DNA with all the combined amplicons that are then incubated with Lipofectamin 3000, before being dispensed equally to each well of a 96 well plate?

Minor comments:
Line 57: Missing "and" between "using vaccinia virus vectors" and "bacterial artificial chromosomes"
Line 345: "observed at 48 pi" should read "observed at 48 hours pi"
LIne 367: Typo in "demontsrtating
Line 292: "After 5 days of transfection", would mean that the transfection was performed over 5 days. It is more likely that the authors means "Five days after transfection"
Reference : lines 580-581 :Infectious RNA transcribed in vitro from a cDNA copy of the human coronavirus genome cloned in vaccinia virus (Thiel et al.) is incorrect an incomplete, see DOI: 10.1099/0022-1317-82-6-1273
Line 470 : "showed either no difference", the word "either" should be removed.

Referee #2:

The Manuscript by Mélade et al, describes an original and rapid reverse genetic procedure useful for rescue infectious coronavirus (CoV) cDNA clones using simple operating conditions. This method has been applied to the rescue of two different CoVs, SARS-CoV-2 and a feline CoV, and different variants of these viruses, and also of subgenomic replicons. The method could be very useful for the selection of antivirals, therapeutic antibodies and vaccine design. A fluorescent marker has been introduced in the recombinant virus to facilitate all these tasks. The system allowed the engineering of Infectious Subgenomic Aplicons (ISA), and is based on the simple co-trasfection of eight overlapping subgenomic DNA fragments covering the full-length genome. To this end, all these cDNAs were transfected into a BHK cells, followed by two additional passages in VERO cells. The DNA fragments were expressed under the control of the cytomegalovirus promoter.

Although a large list of different procedures for the engineering of recombinant CoVs has been reported, as described in the introduction of the manuscript, using either bacteria or yeast, this is the first time that the simultaneous transfection of eight cDNA fragments into mammalian cell is described. Recovered viruses were characterized in tissue culture and in a hamster model in the case of the SARS-CoV-2. Overall, the system seems efficient for the reconstitution de infection mutants or replicons derived from them. Nevertheless, the manuscript strength could be significantly reinforced by, at least, clarifying several items listed under specific points and with a careful review of the paper to correct several minor points.

Specific points

1. Fragment construction. In order to apply the technology used to other viral systems, it would be helpful a description of the length of the overlapping fragments, to achieve an optimum recombination yield, in order to assemble a full-length genome starting from eight independent fragments that need to be precisely link in a single one. Information on the relation between DNA fragments overlapping length and fragment assembly efficiency would also be useful.

2. The assembly of eight different fragments by recombination inside a mammalian cell does not seem an easy task. The incomplete incorporation of all the fragments in 100% of the assembled molecules is a possibility. Obviously, if a fragment corresponds to an essential gene required for virus replication, that would not represent a problem because this incomplete genome will be excluded. Nevertheless, not all viral genes are essential for virus replication, and the theoretical possibility of generation of incomplete genomes is open. Did the author evaluate the proportion of full-length virus recovered in relation to those missing some sequences? As the authors have performed a deep sequence analysis, the data obtained could be used to answer this question. Please, clarify.

3. It is indicated that the SARS-CoV-2 rescued include the furin fragment within the S protein. This is a surprising result, as it is well known that in the VERO cells that have been used for virus rescued it has been described that the furin encoding fragment is deleted in the majority of the recovered viruses. Nevertheless, it seems that the rescued viruses were passed only two times. The question is whether the furin fragment was maintained in the virus with a few additional passages. Please, clarify.

4. Most of the data to analyze recombinant virus rescue are based on the analysis of the gRNA. Please, note that sgmRNA analysis should be performed in addition of gRNA, since gRNA detected by RT-PCR may also result from transcription from CMV promoter, while sgmRNA is only synthesized by a functional CoV replication-transcription complex. In addition, please, note that it is safer to perform plaque assays based on the recovery of viable infectious.

5. As indicated in Table 1, and Fig. 2, even after two passages, titers of the engineered wild type viruses (both for SARS-CoV-2 and FeCoV) are 100-fold lower than those for the original reference virus. Please, clarify. One possibility is that the lower titer of the recombinant viruses could be due to the artificial introduction of point mutations or deletions in the rescued virus. Please, clarify.

6. Virus recombination is cell dependent. Then, it would be highly relevant to indicate in Supplemental Table 3 whether infectious virus was recovered in each of the cell lines used and, that being the case, viral titers could also be indicated.

7. D614G mutant virus decreased viral RNA accumulation and increased hamster weight loss, what seem at variance with previously published data (Hou Y.J. et al, 2020, Science 370:1464-68; Stauft C.B. et al, 2021, Virology 556:96-100). Authors may want to clarify these issues in the discussion.

8. In general, CoVs are reluctant to maintain foreign genes in their genome during passage. Was the mCherry gene inserted in the virus genome stable? In discussion (lines 453-455) it is indicated that this reported gene "appeared to be stable for at least five passages" but no supporting data has been included. In addition, please note that in the study of Rihn et al, 2021, the fluorescent markers were introduced in a different genome location (7a7b genes) and substantial reporter gene loss was found between p3 and p5. Please, comment of this issue.

Minor comments:

1. Figs. 3 and 5. The quality of the images should be improved. One possibility would be to present phase contrast and fluorescence images separately.

2. Fig. 3. Please note that there is a minor error in figure legend. According to the text, it should be "moi of 0.05"

4. Line 441. "G614D" should be "D614G".

5. Please, review carefully the reference list. There is one repeated reference and eight non-completed references. In addition, the format of the references changes along the manuscript, as they appear in the text quoted using numbers of with the name of the first author.

6. Line 57, "or" is missing between "vaccinia virus vectors" and "bacterial...."

7. Table 1, Title. It is missing the table number (1). Similar observation applies to the reference to this table in lines 304, 329, and 348.

8: Lines 358-358. Please, correct spelling and spacing.

9. Line 377. Please, add a point after the reference.

10. Figure 5. A serum negative control may be required.

Referee #3:

Melade et al. established the new reverse genetics system for recombinant coronaviruses using infectious subgenomic amplicons (ISA) technology. The SARS-CoV-2 and the feline enteric coronavirus could be rescued without reconstructing the full-length viral cDNAs.
While this system is simple and useful for coronaviruses, the authors should clarify the advantages of ISA technologies comparing the other similar bacteria-free reverse genetics system for SARS-CoV-2 that have been reported by 2 groups (Torii et al., Cell reports, 2021 and Amarilla et al., Nature commun., 2021).

Major comments:
1. The explanation of de novo synthesis of viral fragments is confusing. The authors should explain what "fragments" (i.e. cDNA fragment, PCR fragment) were supplied (Line 109), what "fragments" were flanked during de novo synthesis (Line 110-113), and what "fragments" were transfected (Line 130). It is also unclear how the pCMV and SV40 sequence were prepared and how the point mutation and the sequence of fluorescent protein were introduced. Were both genes synthesized and supplied by the manufacturer? In addition, using DNA template, which the authors performed, RT-PCR or PCR in Line 127?
2. In Line 287, please clarify the genome recombination. Without ligation enzyme, how is the full-length viral genome assembled efficiently?
3. In Line 340, the authors should illustrate where the mCherry gene was inserted with the nucleotide number.
4. Why the infectious titer from hamsters were higher in the D614 strain than that of the European strain in Fig 6B and C, while its growth is slower than the Eurpean strain in Fig 2A?
5. In Fig 3, we don't know if the amount of virus infected is MOI:0.5 or MOI:0.05, because the authors says MOI:0.5 in Figure legend and MOI:0.05 in main text. In any case, the proportion of mCherry-positive cells appear to be too low, even 48 hours after infection. Please explain this point.
6. As characterization of recombinant virus, evaluation of infectious titer over time and Northern blotting should be performed.

Minor commetns:
1. In Line 32, please write the size of Nidovirales virus genome like as 30 kb.
2. In Line 40, please distinguish two Zhou et al., 2020 papers.
3. In Line 287, please correct "recombination's"
4. In Line 345, please add the correct unit.

Referee #1:

In their manuscript "A simple reverse genetics method to generate recombinant coronaviruses", Mélade et al. describe a method called Infectious Subgenomic Amplicons (ISA), used to rescue two coronaviruses, SARS-CoV-2 and the feline enteric coronavirus. This method had been described before and used by the same group to generate infectious viral clones for several other RNA viruses (e.g.: flavivirus, picornavirus, alphavirus). Although not novel, this method appears to be much simpler and faster to generate recombinant clones of SARS-CoV-2 than all other methods described so far (Thi Nhu Tao et al., 2020, Hou et al., 2020, Rhin et al., 2021 and Xie et al. 2020).

The main findings presented in this article are that 1) the ISA method provides a fast and easy way to rescue SARS-CoV-2 and generate new infectious clones of coronaviruses that have been known to be difficult to rescue and that 2) the infectious SARS-CoV-2 clones produced by ISA are reliable tools to study SARS-CoV-2 biology. These findings are potentially of great interest to the scientific community, in particular to molecular virologists.

However, in its current version, the article does not provide enough information allowing a fair assessment of the work. First, the description of the ISA method itself is unclear. Second, the assessment of the viral clones' genetic stability is limited (cut-off at 50% after 2 passages). Third, the assays used to test the infectious clones generated by ISA are also unclear (see below the Main Comments section). Finally, the authors do not explain how their statistical tests are suited for their experiments (two way ANOVA, Sidak's test correction, "Statistical analysis W"...) which contributes to the difficulty to assess their conclusions.
The manuscript would greatly benefit from improvements and clarification on all these issues.

Main comments:

Genetic stability:
The authors evaluate the genetic stability of their infectious clones generated by ISA, by performing full genome viral sequencing (Ion Torrent) and report only 2 non-synonymous mutations. This is better than what has been reported for other SARS-CoV-2 rescue platforms (Thi Nhu Tao et al., 2020, Silvas et al 2021). However, the authors set their cut off for mutations at 50%. Viral RNA genetic landscapes can be very diverse and mutations under 50% that can easily hide important viral genetic heterogeneity. Since the authors only performed the sequencing experiment once, it is important that they provide the sequencing results and more importantly report all mutations location and nature (synonymous and non-synonymous), represented in at least 10% of the population.
We agree with the reviewer's comment and we followed the recommendation. We added in the new version of the manuscript the sequencing results for mutation recorded with frequency between 5% to 50%, in Table 2 and we described the results along the revised version of the manuscript. We also defined those mutations as « minority mutations ». We also added the location and nature for all mutations recorded.

Neutralizing antibody and antiviral assays:
The method for the fluorescent based neutralization assay is not clear. Also the authors mention "dose-response curves generated using GraphPad Prism 7.00 with a four parameters linear regression model." but they are not shown and their purpose is unclear. The author should describe their negative control for fluorescent, the exact number of areas scanned per well and for each condition and what was considered as an absence of fluorescence for neutralization assay. Similarly,

what is the reference signal for the percentage of fluorescence signal in the y axis in Figure 4b?
Figure 5: the legend says that the "EC50 values presented are from three and two independent experiments respectively". However, the figure shows 5 data points for each series at each concentration.
We agree with the reviewer's comment. A negative control was added in which, the sera were pre-incubated with a non-fluorescent SARS-CoV-2 strain (ISA D614 SARS-CoV-2) as described previously and added onto confluent monolayers of Vero-E6 cells for 5 days at 37°C with 5% CO2. We ensured that no fluorescence was observed in this control. This information was added in lines 204-208 in the revised manuscript. The quantification of the fluorescence was performed following the manufacturer recommendation: one randomly selected area in the well was analyzed (this corresponds to approximately 1/9 of the well). The fluorescent cells % obtained in non-infected wells ranged between 0% to 0.3%. Wells were considered positive when at least 1% of infected cells were recorded fluorescent by the reader and this value was considered as reference for positive fluorescent wells and used to set up the threshold of positivity.

Regarding EC50: we thank the reviewer for the comment and agree that our representation could be confusing for the reader. We indeed pooled two experiments in triplicate (n=6) in the same graph. In order to improve the manuscript, we amended it by presenting the result of one representative experiment for the dose response curve. In addition, the calculated EC50 are from two independent experiments with the two read-outs done in parallel, in order to compare "apple to apple". We modified the corresponding values accordingly as well as the legend of the figure: "a: Dose response curve for the ISA D614 and for the mCherry D614 strains obtained by fluorescence or viral RNA measurement in VeroE6 cells from one representative experiment. Each point represents the mean ± SD of a triplicate. b: Table of EC50 values obtained for the two different strains from two independent experiments. c: Fluorescence of the SARS-CoV-2 mCherry in VeroE6 cells with different Remdesivir concentration. EC50: 50% inhibition, Remdesivir concentrations are presented in log scale for logarithmic interpolation. Dose response curves were generated using GraphPad Prism software version 7.0 (https://graphpad-prism.software.informer.com/7.0/)"

ISA method:
In their method section, the authors describe using "A equimolar mix (300ng) of subgenomic cDNA fragments of SARS-CoV-2" (line 132). It is unclear if they mean a total of 300ng cDNA for the combined 8 amplicons reconstituting the viral genome or that 300ng of each amplicon was added to the transfection mix per well. In line 430, they say that the ISA experiment is performed using a "a total of ~30 µg of mixed PCR fragments" in a 96 well plate. Does it mean that the ISA is actually performed using a total of 30ug DNA with all the combined amplicons that are then incubated with Lipofectamin 3000, before being dispensed equally to each well of a 96 well plate?
We thank the reviewer for pointing this out. Indeed, the one-transfection assay is performed with a quantity of 300 ng of the eight pooled DNA fragments (around 25 ng to 55 ng of each fragments, depending on fragment length). We indicated this in line 140-141 in revised the manuscript. The protocol indicated in the Material and Methods is for 1 well of a 96 well plate. In our side, all eight fragments (final quantity of 30 µg (300ng *96 wells) were combined together and mixed with Lipofectamine 3000 and distributed into each wells of the 96 wells plate.

Minor comments:
Line 57: Missing "and" between "using vaccinia virus vectors" and "bacterial artificial chromosomes"

Line 345: "observed at 48 pi" should read "observed at 48 hours pi"
LIne 367: Typo in "demontsrtating
Line 292: "After 5 days of transfection", would mean that the transfection was performed over 5 days. It is more likely that the authors means "Five days after transfection"
Reference : lines 580-581 :Infectious RNA transcribed in vitro from a cDNA copy of the human coronavirus genome cloned in vaccinia virus (Thiel et al.) is incorrect an incomplete, see DOI: 10.1099/0022-1317-82-6-1273
Line 470 : "showed either no difference", the word "either" should be removed.
We thank the reviewer for pointing out the grammar and typing mistakes. We performed corrections along the manuscript according to reviewer's recommendations.

Referee #2:
The Manuscript by Mélade et al, describes an original and rapid reverse genetic procedure useful for rescue infectious coronavirus (CoV) cDNA clones using simple operating conditions. This method has been applied to the rescue of two different CoVs, SARS-CoV-2 and a feline CoV, and different variants of these viruses, and also of subgenomic replicons. The method could be very useful for the selection of antivirals, therapeutic antibodies and vaccine design. A fluorescent marker has been introduced in the recombinant virus to facilitate all these tasks. The system allowed the engineering of Infectious Subgenomic Aplicons (ISA), and is based on the simple co-trasfection of eight overlapping subgenomic DNA fragments covering the full-length genome. To this end, all these cDNAs were transfected into a BHK cells, followed by two additional passages in VERO cells. The DNA fragments were expressed under the control of the cytomegalovirus promoter.

Although a large list of different procedures for the engineering of recombinant CoVs has been reported, as described in the introduction of the manuscript, using either bacteria or yeast, this is the first time that the simultaneous transfection of eight cDNA fragments into mammalian cell is described. Recovered viruses were characterized in tissue culture and in a hamster model in the case of the SARS-CoV-2. Overall, the system seems efficient for the reconstitution de infection mutants or replicons derived from them. Nevertheless, the manuscript strength could be significantly reinforced by, at least, clarifying several items listed under specific points and with a careful review of the paper to correct several minor points.

Specific points

1. Fragment construction. In order to apply the technology used to other viral systems, it would be helpful a description of the length of the overlapping fragments, to achieve an optimum recombination yield, in order to assemble a full-length genome starting from eight independent fragments that need to be precisely link in a single one. Information on the relation between DNA fragments overlapping length and fragment assembly efficiency would also be useful.
We understand the reviewer's question regarding the optimum recombination yield and the size or overlap of DNA fragments. In this study, infectious particles were rescue using a "standard" procedure with optimized conditions as used in previous publications: fragment length of around 4kb and overlapping region size up to 50bp are generally synthetized. We added the precise size of each fragments and overlapping region on supplemental Table 1. In previous attempts, we added the promotor (pCMV) and terminator (HDR/SV40pA) separately, as additional fragments and this lead to a total of 10 overlapping cDNA fragments. However, the size of the first and last fragments were decreased. Although this method has already shown its capacity to rescue small ssRNA viruses, in our study, we could not rescue coronaviruses with this method. Thus we assumed that the number of fragment is a crucial parameter to achieve recombination and generation of infectious coronavirus particles than the size of fragments themselves.

2. The assembly of eight different fragments by recombination inside a mammalian cell does not seem an easy task. The incomplete incorporation of all the fragments in 100% of the assembled molecules is a possibility. Obviously, if a fragment corresponds to an essential gene required for virus replication, that would not represent a problem because this incomplete genome will be excluded. Nevertheless, not all viral genes are essential for virus replication, and the theoretical possibility of

generation of incomplete genomes is open. Did the author evaluate the proportion of full-length virus recovered in relation to those missing some sequences? As the authors have performed a deep sequence analysis, the data obtained could be used to answer this question. Please, clarify.
The reviewer comment is relevant. We did performed a deep sequencing to determine the mutations found with frequencies between 5% - 50% in sequencing reads, defined as minority mutations in this study. The results were added now in Table 2. Few minority mutations were recorded in the rescued wild-type and recombinant viruses. No deletion was observed in the complete genome of each rescued viruses.

3. It is indicated that the SARS-CoV-2 rescued include the furin fragment within the S protein. This is a surprising result, as it is well known that in the VERO cells that have been used for virus rescued it has been described that the furin encoding fragment is deleted in the majority of the recovered viruses. Nevertheless, it seems that the rescued viruses were passed only two times. The question is whether the furin fragment was maintained in the virus with a few additional passages. Please, clarify.
We agree with the reviewer. Depending on the cell line, a furin deletion can commonly occurr. This is particularly observed in Vero E6 in which TMPRSS2 is not or poorly expressed. We followed the reviewer recommendation and additionally performed 3 supplemental passages on VeroE5 cells (5 passages in total) and look for deletion in the complete genome of each recombinant viruses and particularly in the spike protein using NGS. No deletion was observed in each recombinant SARS-CoV-2 strains after a total of 5 passages. We added this supplemental information in the results section in our manuscript (lines 480-485).

4. Most of the data to analyze recombinant virus rescue are based on the analysis of the gRNA. Please, note that sgmRNA analysis should be performed in addition of gRNA, since gRNA detected by RT-PCR may also result from transcription from CMV promoter, while sgmRNA is only synthesized by a functional CoV replication-transcription complex. In addition, please, note that it is safer to perform plaque assays based on the recovery of viable infectious.
We agree with the recommendation particularly for SARS-CoV-2 recombinants for which *in vitro* experiments, seoneutralization and antivirals assays were performed. We used a sub genomic detection system developed and published by Wölfel et al 2020 (PMID: 32235945) to quantify the subgenomic RNA for the E gene in the cell lysate of each recombinant SARS-CoV-2 viruses after 2 passages and added supplemental information in the Material and Methods section (lines 268-270). As seen in supplemental table 6, the subgenomic RNA quantification for the E gene was confirmed for all clinical and recombinant SARS-CoV-2 strains. In addition to supplemental table 6, the results section has been amended.

We also agree that plaque assay should be useful and help to confirm the identical phenotype in cell culture. However, we used the combination of phenotypic parameters such as CPE, TCID50, and kinetics growth allow us to ensure that the rescue virus display similar phenotype *in vitro* and all results confirmed the similarity between rescued strain and clinical isolate.

5. As indicated in Table 1, and Fig. 2, even after two passages, titers of the engineered wild type viruses (both for SARS-CoV-2 and FeCoV) are 100-fold lower than those for the original reference virus. Please, clarify. One possibility is that the lower titer of the recombinant viruses could be due to

the artificial introduction of point mutations or deletions in the rescued virus. Please, clarify.
We agree with the reviewer. This lower virulence could be the consequence of viral adaptation, such as the appearance of mutations and quasispecies, which may promote the replicative fitness of the rescued virus. It is recognized that clinical strains which are behaving as quasispecies, are better adapted to their environment (PMID: 22688811) in comparison to clonal or quasi-clonal viruses. We would argue that the reconstruction of wild type SARS-CoV-2 and FeCoV was performed with DNA synthetic DNA fragments exempt of artificial mutation or deletion and cDNA fragments were submitted to sequencing before transfection to ensure their genetic integrity.

6. Virus recombination is cell dependent. Then, it would be highly relevant to indicate in Supplemental Table 3 whether infectious virus was recovered in each of the cell lines used and, that being the case, viral titers could also be indicated.
We understand the reviewer's remark. However, so far, the only cell line or combination of cell lines used to rescue infectious and viable SARS-CoV-2 was the one described in the material and method of this study. Several attempts were performed using numerous cell lines and combination of cell lines described in Supplemental 3, but, with our conditions, infectious viruses were rescue only when BHK-21 where transfected and VeroE6 cells were added after 24 hours.

7. D614G mutant virus decreased viral RNA accumulation and increased hamster weight loss, what seem at variance with previously published data (Hou Y.J. et al, 2020, Science 370:1464-68; Stauft C.B. et al, 2021, Virology 556:96-100). Authors may want to clarify these issues in the discussion.
The reviewer remark is relevant. The effect of the D or G614 in the spike protein is complex in the hamster model. Hou Y.J. et al 2020, indicated (page 2): "However, the D614G-infected hamsters lost slightly more body weight than those infected with the WT virus (Fig. 4C)."In their study, Stauft C.B. et al, 2021, showed that clinical D614 virulence was similar to clinical G614 strain as cited (page 98) "on average hamsters in both groups lost up to 14% body weight in 7 days, however the amount of weight loss was comparable for both D614 and G614 over the course of two weeks no difference in the loss of weight between the two strains (Fig. 2A)". This was similar to the study of Plante et al., 2020 (PMID: 32908978) as cited (page 3): "Four- to five-week-old hamsters were infected intranasally with 2 × 104 PFU of D614 or G614 virus. Infected hamsters from both groups exhibited similar weight loss (Fig. 2a).

In our study, we observed that clinical G614 (European) and rescued D614 exhibited the same virulence as recorded by the similar weight loss. This is in accordance with Stauft C.B. et al, 2021 and Plante et al., 2020. Of note, the virulence of the rescued G614 (European) was lower than the rescued D614 but more importantly, lower than the clinical isolate G614 (European) as illustrated by the lower loss of weight in hamster. *In vivo* differences in viral fitness between reverse genetic based virus and clinical virus has already been observed particularly for several ISA strains (PMID: 25734338). As already mentioned regarding the *in vitro* results, this can be the consequence of an adaptation of the rescue strain to the cell culture environment in comparison to a well-adapted clinical strain (PMID: 22688811). This hypothesis is well supported regarding the genotypic changes (majority and minority mutations) of the rescue G614 strain we observed after 2 passages in cell culture.

8. In general, CoVs are reluctant to maintain foreign genes in their genome during passage. Was the

mCherry gene inserted in the virus genome stable? In discussion (lines 453-455) it is indicated that this reported gene "appeared to be stable for at least five passages" but no supporting data has been included. In addition, please note that in the study of Rihn et al, 2021, the fluorescent markers were introduced in a different genome location (7a7b genes) and substantial reporter gene loss was found between p3 and p5. Please, comment of this issue.
The reviewer comment is fair. The insertion of reporter gene is hardly maintained on coronavirus genome and as described by Rihn et al 2021, a deletion was observed. In our study the mCherry was inserted into the 3a accessory protein and not in the 7ab accessory protein. To assess the stability of the mCherry in the viral genome of our recombinant strain, the rescued virus was passaged 3 more times on VeroE6 cells. At each passages, the presence of the mCherry was verified using a RT-PCR system (lines 157-163 in Material and methods), analysis on agarose gel (Figure 3b) and sequenced by NGS for mutations. As observed on agarose gel after electrophoresis, the mCherry reporter gene was present at any passage. Sequencing results confirmed the total integrity of the introduced gene with no majority and minority mutations after 5 passages. The results were added in lines 395-398 in the results section of the manuscript.

Minor comments:

1. Figs. 3 and 5. The quality of the images should be improved. One possibility would be to present phase contrast and fluorescence images separately.
We improved the quality of the picture through Incucyte output files parameters to improve all pictures luminosity and provide high quality figures (600dpi).

2. Fig. 3. Please note that there is a minor error in figure legend. According to the text, it should be "moi of 0.05".
We corrected the text.

4. Line 441. "G614D" should be "D614G".
To avoid confusion and harmonize the text, we replaced the name here by “ISA D614” strain

5. Please, review carefully the reference list. There is one repeated reference and eight non-completed references. In addition, the format of the references changes along the manuscript, as they appear in the text quoted using numbers of with the name of the first author.
The reference list was corrected and the number in the text were replaced .

6. Line 57, "or" is missing between "vaccinia virus vectors" and "bacterial...."
Corrected.

7. Table 1, Title. It is missing the table number (1). Similar observation applies to the reference to this table in lines 304, 329, and 348.
Corrected.

8: Lines 358-358. Please, correct spelling and spacing.
Corrected.

9. Line 377. Please, add a point after the reference.
Thank you, corrected.

10. Figure 5. A serum negative control may be required.

We believe that reviewer was referring to Figure 4. In this study we used and tested 6 sera that were known to be negative for SARS-CoV-2 antibodies. These sera were thus used as negative control.

Referee #3:

Melade et al. established the new reverse genetics system for recombinant coronaviruses using infectious subgenomic amplicons (ISA) technology. The SARS-CoV-2 and the feline enteric coronavirus could be rescued without reconstructing the full-length viral cDNAs.
While this system is simple and useful for coronaviruses, the authors should clarify the advantages of ISA technologies comparing the other similar bacteria-free reverse genetics system for SARS-CoV-2 that have been reported by 2 groups (Torii et al., Cell reports, 2021 and Amarilla et al., Nature commun., 2021).

Major comments:
1. The explanation of de novo synthesis of viral fragments is confusing. The authors should explain what "fragments" (i.e. cDNA fragment, PCR fragment) were supplied (Line 109), what "fragments" were flanked during de novo synthesis (Line 110-113), and what "fragments" were transfected (Line 130). It is also unclear how the pCMV and SV40 sequence were prepared and how the point mutation and the sequence of fluorescent protein were introduced. Were both genes synthesized and supplied by the manufacturer? In addition, using DNA template, which the authors performed, RT-PCR or PCR in Line 127?
We thanks the reviewer for this comment. Based on the reviewer's comment, we brought modifications to clarify the difference between synthetic *de novo* fragments and cDNA fragments produced by PCR for the transfection. During the bioinformatic design of the DNA fragments, we directly flanked the promotor and terminator sequence upstream and downstream of the first and last fragment respectively. No additional manipulation was necessary. The process was similar for mutagenesis or addition of the red fluorescent protein gene. Yes, the fragment with the promotor, terminator, mutation and red fluorescent protein were directly provided by the manufacturer. We added clarification about this in lines 308-312 in the revised version of the manuscript.

2. In Line 287, please clarify the genome recombination. Without ligation enzyme, how is the full-length viral genome assembled efficiently?
The remark is relevant. The *in cellula* recombination process and how fragments are reassembled to generate a fully functional complete genome are not clearly elucidated to date. Because of CMV has a nuclear activity, we assume that preliminary mechanism such as transcription, splicing and mRNA production occurs at the nuclear level of cells. We previously applied the ISA method in a large range of mammalian cell lines and in mosquito cells (PMID: 25053561; PMID: 29070887). Thus, several process seems to occur in many cell lines and probably involve conserved biological properties. Unfortunately, we cannot advance further hypothesis.

3. In Line 340, the authors should illustrate where the mCherry gene was inserted with the nucleotide number.
We thank the reviewer for this remark and add the information in lines 123-126 and 379-380 in the revised manuscript.

4. Why the infectious titer from hamsters were higher in the D614 strain than that of the European strain in Fig 6B and C, while its growth is slower than the Eurpean strain in Fig 2A?
The point raised by the reviewer is relevant. Indeed, the results obtained *in vivo* and *in vitro* showed discrepancy for the ISA D614 and the ISA European strain. Particularity the *in vivo* experiments showed that virulence of the rescued G614 (European) was lower than the rescued D614 but more importantly, lower than the clinical isolate G614 (European) as illustrated by the lower loss of weight

in hamster. *In vivo* differences in viral fitness between reverse genetic based virus and clinical virus has already been observed particularly for several ISA strains (PMID: 25734338). We thus believed that the discrepancy observed *in vivo* can be the consequence of an adaptation of the rescue strain to the cell culture environment in comparison to a well-adapted clinical strain (PMID: 22688811) and thus are not in concordance with the in vitro results. This hypothesis is well supported regarding the genotypic changes (majority and minority mutations) of the rescue G614 strain we observed after 2 passages in cell culture

5. In Fig 3, we don't know if the amount of virus infected is MOI:0.5 or MOI:0.05, because the authors says MOI:0.5 in Figure legend and MOI:0.05 in main text. In any case, the proportion of mCherry-positive cells appear to be too low, even 48 hours after infection. Please explain this point.
We apologize for this typing error. The MOI used here was indeed 0.05. We agree the number of fluorescent is low, but as long as we can correlate the level of fluorescence with the seroneutralization effect, we think that the assay is relevant as such.

6. As characterization of recombinant virus, evaluation of infectious titer over time and Northern blotting should be performed.

We thank the reviewer for this comment. Indeed, infectious titer may be relevant to analyzed the kinetic growth. Therefore, we followed the reviewer recommendation and added supplemental results (Figure 2b,d) to described the kinetics growth using the TCID50 method. The results obtained by TCID50 method and RT-qPCR method were added along the results section of the manuscript and in the discussion section. We additionally designed 4 RT-PCR systems to detected RNA derived from the spike, membrane, nucleocapsid and ORF7a. As it is beyond our expertise to perform Northern Blot and regarding the difficulties to ship our sample to other lab we decided to positively answer this by performing this experiment. We added the results of the experiments in supplemental Figure 1 and discuss the results section in the revised manuscript. In complementary results, we used a sub genomic detection system developed and published by Wölfel et al 2020 (PMID: 32235945) to quantify the subgenomic RNA for the E gene in the cell lysate of each recombinant SARS-CoV-2 viruses after 2 passages and added supplemental information in the Material and Methods section (lines 268-270).

Minor commetns:
1. In Line 32, please write the size of Nidovirales virus genome like as 30 kb.
The information was added.

2. In Line 40, please distinguish two Zhou et al., 2020 papers.
The modification was performed.

3. In Line 287, please correct "recombination's"
Correction was performed.

4. In Line 345, please add the correct unit.
The correct unit was added.

Dear Dr. Mélade,

Thank you for the submission of your revised manuscript to our editorial offices. I have received the report from three referees that were asked to re-evaluate your study, you will find below. As you will see, the referees now support the publication of your study. Nevertheless, referees #2 and #3 have remaining concerns and suggestions to improve the manuscript, I ask you to address in a final revised version of the paper. Please also provide a point-by-point response addressing the remaining points of the referees.

Please also make sure that all the key information provided has been included in the final manuscript text (see point 2 of referee #2) and that the novelty of the approach and advantages of this method compared to similar bacteria-free reverse genetics approaches is appropriately highlighted in the introduction and the discussion (referee #3).

Moreover, I have these editorial requests I also ask you to address:

- Please add up to five keywords to the title page.

- Please provide the abstract written in present tense.

- Per journal policy, we do not allow 'data not shown', which is stated in the manuscript (page 17). All data referred to in the paper should be displayed in the main or Expanded View figures, or an Appendix. Thus, please add these data (or change the text accordingly if these data are not important). See:
http://embor.embopress.org/authorguide#unpublisheddata

- We plan to publish your manuscript in the Report format with up to 5 main figures. For a Scientific Report we require that results and discussion sections are combined in a single chapter called "Results & Discussion". Please do this for your manuscript. For more details please refer to our guide to authors:
http://www.embopress.org/page/journal/14693178/authorguide#researcharticleguide

- Please combine the data shown in the six main figures into 5 final main figures. If possible, please also add what is presently shown in the two supplementary figures to the main figures. I think it will be possible show all these data in 5 main figures. We need these as individual production quality figure files as .eps, .tif, .jpg (one file per figure). Please upload these as separate, individual files upon re-submission. The final text file should not contain any figures or supplementary information.

Please consult our guide for figure preparation:
http://wol-prod-cdn.literatumonline.com/pb-assets/embo-site/EMBOPress_Figure_Guidelines_061115-1561436025777.pdf

See also the guidelines for figure legend preparation:
https://www.embopress.org/page/journal/14693178/authorguide#figureformat

- The Expanded View format, which will be displayed in the main HTML of the paper in a collapsible format, has replaced the Supplementary information. Thus, if you feel that the data shown in the Supplementary figures should not be combined with the main figures (as suggested above), these should be submitted as Expanded View (again one file per figure; individual production quality figure files as .eps, .tif, .jpg). Please follow the nomenclature Figure EV1, Figure EV2 etc. The figure legend for these should be included in the main manuscript document file in a section called Expanded View Figure Legends after the main Figure Legends section.

- Please add a 'Data Availability' section (DAS) to the manuscript, also if no large datasets have been submitted to a public database. Please state there 'No large primary datasets have been generated and deposited'. Further, please indicate this clearly in the author checklist (field F18).

- Please completely fill in the author checklist, in particular fields D10 and G22.

- Please make sure that the number "n" for how many independent experiments were performed, their nature (biological versus technical replicates), the bars and error bars (e.g. SEM, SD) and the test used to calculate p-values is indicated in the respective figure legends (main and EV figures), and that statistical testing has been done where applicable. Please avoid phrases like 'independent experiment', but clearly state if these were biological or technical replicates. Please add complete statistical testing to all diagrams. Please also indicate (e.g. with n.s.) if testing was performed, but the differences are not significant. Presently some diagrams miss statistics or show only partial statistics.

- Please add scale bars of similar style and thickness to the microscopic images, using clearly visible black or white bars (depending on the background). Please place these in the lower right corner of the images. Please do not write on or near the bars in the image but define the size in the respective figure legend.

- I would suggest moving all the Supplementary tables into an Appendix file, as these do not contain main data. Please upload these in one pdf file named 'Appendix' with page numbers and use 'Appendix Table S#' to name the tables. Please use this also as callout in the manuscript text and update all callouts. Please make sure that all tables are called out. Please add a table of contents to the Appendix including page numbers and also add titles and legends to the tables. Finally, please remove the Supplementary tables from the main manuscript file.

- Please include the funding information into the Acknowledgements and make sure that all the funding information is also entered into the online submission system and is complete and similar to the one in the manuscript text file.

- Please remove the section 'Supplementary Data - Supplementary Data are provided online' from the manuscript text.

- Please add a paragraph describing the author contributions to the manuscript text and order the manuscript sections like this (using these section names):
Title page - Abstract - Introduction - Results - Discussion - Materials and Methods - DAS - Acknowledgements - Author contributions - Conflict of interest - References - Figure legends - Expanded View Figure legends (if necessary) - Tables with legends

- Finally, please find attached a word file of the manuscript text (provided by our publisher - without the figures and tables) with a few changes and queries we ask you to include in your final manuscript text. Please provide your final manuscript file with track changes, in order that we can see any modifications done.

In addition, I would need from you:
- a short, two-sentence summary of the manuscript (not more than 35 words).
- two to four short bullet points highlighting the key findings of your study.
- a schematic summary figure (in jpeg or tiff format with the exact width of 550 pixels and a height of not more than 400 pixels) that can be used as a visual synopsis on our website.

I look forward to seeing the final revised version of your manuscript when it is ready. Please let me know if you have questions regarding the revision.

Best,

Achim Breiling
Senior Editor
EMBO Reports

Referee #1:

The authors have address the most important points. It is now appropriate for publication.

Referee #2:

This is the evaluation of the reply of the authors to the comments by three previous reviewers. In this second review, we acknowledge that the authors have made a considerable effort for improvement attending to all the reviewers' suggestions. However, there are still a few issues that would require additional clarification:

1. Answer to Reviewer #2 point 4 and Reviewer #3 point 6. Please note that subgenomic mRNA was not detected following the protocol described in Wölfel et al, 2020, the indicated one. The protocol used is the one described in Corman V.M. et al, 2020, EuroSurveill. 25:2000045, which uses gene E for detection. Due to the CoV transcription mechanism, both gRNA and sgmRNAs for S, 3 and E were detected using this assay and a significant portion of the detected RNA can come from CMV promoter transcription, and not from the viral transcription regulatory sequences. Therefore, the question on specific sgmRNA analysis to confirm a functional replication-transcription complex still is an open one.

2. It is not clear from the reply to the reviewers whether key information provided has been included in the new manuscript. For instance, information on the required fragment design or cell system to succeed, or additional discussion on virus stability.

3. Reply to Reviewer #2 point 5. Please note that virulence was never analyzed, as no in vivo experiment was performed.

Therefore, we would like to suggest, at least to properly complete the first point before full acceptance.

Referee #3:

I think the author has answered each comment carefully. However, the authors have not responded to the advantages of this method compared to similar Bacteria-free reverse genetics(Torii et al., Cell reports, 2021 and Amarilla et al., Nature commun., 2021). At present, it is not considered novel enough to be published in EMBO Reports.

Dear Dr. Mélade,

Thank you for the submission of your revised manuscript to our editorial offices. I have received the report from three referees that were asked to re-evaluate your study, you will find below. As you will see, the referees now support the publication of your study. Nevertheless, referees #2 and #3 have remaining concerns and suggestions to improve the manuscript, I ask you to address in a final revised version of the paper. Please also provide a point-by-point response addressing the remaining points of the referees.

Please also make sure that all the key information provided has been included in the final manuscript text (see point 2 of referee #2) and that the novelty of the approach and advantages of this method compared to similar bacteria-free reverse genetics approaches is appropriately highlighted in the introduction and the discussion (referee #3).
We provided all key information's as requested and indicated in the introduction (line 91) and discussion (line 333) the novelty of the approach and advantages of the ISA method compared to similar bacteria-free reverse genetics approaches.

Moreover, I have these editorial requests I also ask you to address:
- Please add up to five keywords to the title page.
Keywords were added

- Please provide the abstract written in present tense.
The abstract is now write in present tense.

- Per journal policy, we do not allow 'data not shown', which is stated in the manuscript (page 17). All data referred to in the paper should be displayed in the main or Expanded View figures, or an Appendix. Thus, please add these data (or change the text accordingly if these data are not important). See:
http://embor.embopress.org/authorguide#unpublisheddata
As these data are not relevant, we removed the "data not shown".

- We plan to publish your manuscript in the Report format with up to 5 main figures. For a Scientific Report we require that results and discussion sections are combined in a single chapter called "Results & Discussion". Please do this for your manuscript. For more details please refer to our guide to authors: http://www.embopress.org/page/journal/14693178/authorguide#researcharticleguide
The modification was done.

- Please combine the data shown in the six main figures into 5 final main figures. If possible, please also add what is presently shown in the two supplementary figures to the main figures. I think it will be possible show all these data in 5 main figures. We need these as individual production quality figure files as .eps, .tif, .jpg (one file per figure). Please upload

these as separate, individual files upon re-submission. The final text file should not contain any figures or supplementary information.
Please consult our guide for figure preparation:
http://wol-prod-cdn.literatumonline.com/pb-assets/embo-site/EMBOPress_Figure_Guidelines_061115-1561436025777.pdf
See also the guidelines for figure legend preparation: https://www.embopress.org/page/journal/14693178/authorguide#figureformat
We combined the fluorescence results in figure 3 and 4 into one singular figure to obtained a total of 5 final figures.

- The Expanded View format, which will be displayed in the main HTML of the paper in a collapsible format, has replaced the Supplementary information. Thus, if you feel that the data shown in the Supplementary figures should not be combined with the main figures (as suggested above), these should be submitted as Expanded View (again one file per figure; individual production quality figure files as .eps, .tif, .jpg). Please follow the nomenclature Figure EV1, Figure EV2 etc. The figure legend for these should be included in the main manuscript document file in a section called Expanded View Figure Legends after the main Figure Legends section.
We keep the actual format for the 2 supplemental figures and modified the nomenclature in the legend and in the manuscript file.

- Please add a 'Data Availability' section (DAS) to the manuscript, also if no large datasets have been submitted to a public database. Please state there 'No large primary datasets have been generated and deposited'. Further, please indicate this clearly in the author checklist (field F18).

We added this section.

- Please completely fill in the author checklist, in particular fields D10 and G22.
We filled the document.

- Please make sure that the number "n" for how many independent experiments were performed, their nature (biological versus technical replicates), the bars and error bars (e.g. SEM, SD) and the test used to calculate p-values is indicated in the respective figure legends (main and EV figures), and that statistical testing has been done where applicable. Please avoid phrases like 'independent experiment', but clearly state if these were biological or technical replicates. Please add complete statistical testing to all diagrams. Please also indicate (e.g. with n.s.) if testing was performed, but the differences are not significant. Presently some diagrams miss statistics or show only partial statistics.
We added supplemental information's in the figure legends and modified the different figures according to the recommendation.

- Please add scale bars of similar style and thickness to the microscopic images, using clearly visible black or white bars (depending on the background). Please place these in the lower right corner of the images. Please do not write on or near the bars in the image but define the size in the respective figure legend.
We added scale bar for the microscopic picture.

- I would suggest moving all the Supplementary tables into an Appendix file, as these do not contain main data. Please upload these in one pdf file named 'Appendix' with page numbers and use 'Appendix Table S#' to name the tables. Please use this also as callout in the manuscript text and update all callouts. Please make sure that all tables are called out. Please add a table of contents to the Appendix including page numbers and also add titles and legends to the tables. Finally, please remove the Supplementary tables from the main manuscript file.
We followed the recommendation and moved all the supplemental tables into an appendix file with the recommendation.

- Please include the funding information into the Acknowledgements and make sure that all the funding information is also entered into the online submission system and is complete and similar to the one in the manuscript text file.
We added the funding information in the acknowledgements section and thought online submission.

- Please remove the section 'Supplementary Data - Supplementary Data are provided online' from the manuscript text.
The section was removed.

- Please add a paragraph describing the author contributions to the manuscript text and order the manuscript sections like this (using these section names):
Title page - Abstract - Introduction - Results - Discussion - Materials and Methods - DAS - Acknowledgements - Author contributions - Conflict of interest - References - Figure legends - Expanded View Figure legends (if necessary) - Tables with legends

We modified the manuscript structure following the recommendation.

- Finally, please find attached a word file of the manuscript text (provided by our publisher - without the figures and tables) with a few changes and queries we ask you to include in your final manuscript text. Please provide your final manuscript file with track changes, in order that we can see any modifications done.
We followed the recommendation.

In addition, I would need from you:
- a short, two-sentence summary of the manuscript (not more than 35 words).
- two to four short bullet points highlighting the key findings of your study.
- a schematic summary figure (in jpeg or tiff format with the exact width of 550 pixels and a height of not more than 400 pixels) that can be used as a visual synopsis on our website.
These supplemental information's were indicated in the title page of the manuscript.

I look forward to seeing the final revised version of your manuscript when it is ready. Please let me know if you have questions regarding the revision.

Best,

Achim Breiling
Senior Editor
EMBO Reports

Referee #1:

The authors have address the most important points. It is now appropriate for publication.

Referee #2:

This is the evaluation of the reply of the authors to the comments by three previous reviewers. In this second review, we acknowledge that the authors have made a considerable effort for improvement attending to all the reviewers' suggestions. However, there are still a few issues that would require additional clarification:

1. Answer to Reviewer #2 point 4 and Reviewer #3 point 6. Please note that subgenomic mRNA was not detected following the protocol described in Wölfel et al, 2020, the indicated one. The protocol used is the one described in Corman V.M. et al, 2020, EuroSurveill. 25:2000045, which uses gene E for detection. Due to the CoV transcription mechanism, both gRNA and sgmRNAs for S, 3 and E were detected using this assay and a significant portion of the detected RNA can come from CMV promoter transcription, and not from the viral transcription regulatory sequences. Therefore, the question on specific sgmRNA analysis to confirm a functional replication-transcription complex still is an open one.

As mentioned in our previous response, we used the system with leader specific primer that was described by Wolfel et al 2020 publish in Nature journal to specifically detect subgenomic RNAs. As stated in their material and method section on the supplementary information from Wolfel et al 2020:

Section 1. Paragraph b: RT-PCR for subgenomic RNA for SARS-CoV-2:

“Testing for sgRNA used a leader-specific primer, as well as primers and probes targeting sequences downstream of the start codons of the E gene, as described (Corman, Landt et al.2020). In addition to real-time RT-PCR readouts, RT-PCR products for sgRNAs were analyzed on agarose gel and Sanger sequenced (Figure S1). The oligonucleotide sequence of the leader specific primer was as follows: sgLeadSARSCoV2-F; CGATCTCTTGTAGATCTGTTCTC.”

We apologize if the information was not properly presented in the manuscript and we modified it (line 525) and added the sequences information in appendix Table 5 it in order to clearly show that the assay specifically detects sgRNA, using a method identical to the one presented in Wolfe et al. : Additionally, for SARS-CoV-2, the subgenomic (sgm) expression of the E gene was analyzed and quantified following Wölfel et al 2020 (Wölfel et al, 2020) with the sequence of the leader specific, reverse and probe sequences targeting the E gene described in appendix Table 5.

2. It is not clear from the reply to the reviewers whether key information provided has been included in the new manuscript. For instance, information on the required fragment design or cell system to succeed, or additional discussion on virus stability.

We carefully double-checked the previous reviewer comments point by point particularly their recommendations regarding details of the method to achieve transfection success or the hypothesis regarding the virus stability.

> Reviewer 1: modifications regarding the design of the fragments were already added in the previous revised version of the manuscript.

>Reviewer 2: we now added supplemental information regarding:

-the cell lines used (point 4): (line 402) "these cell lines mentioned above were the first that gave recombinant infectious viruses after optimizing a panel of conditions in parallel (Appendix Table 3) and were thus selected for the rest of the study. "

>Reviewer 2 (point 7) and Reviewer 3 (point 4): We indicate now in the new version of the manuscript that (line 319): "*D*ifferences in viral fitness observed *In vivo* between reverse genetics-based virus and clinical virus have already been observed particularly for several ISA strains (PMID: 25734338). This can be the consequence of an adaptation of the rescued strain to the environment in comparison to a well-adapted clinical strain, as also observed for the results observed *in vitro* (PMID: 22688811). This hypothesis is well supported regarding the genotypic changes (majority and minority mutations) of the rescue ISA European strain we observed after 2 passages in cell culture."

The fragment position, size, overlapping size for the design were already added in supplementary table 1. We believe this information is suitable for the reproduction of the experiment.

Additional remarks regarding the *in vitro* virus stability were already added in the previous revised version of the manuscript: for the viral stability produced by the described method, we indicated in the previous revised version of the manuscript (line 278):" the infectious supernatant medium of each rescued strain after 2 passages on VeroE6 cells was additionally passaged 3 more times to determine whether or not additional deletions in the complete genome arose, particularly in the spike protein and indicated a conserved genomic structure.

We removed the "data not shown" as recommended by the journal.

3. Reply to Reviewer #2 point 5. Please note that virulence was never analyzed, as no in vivo experiment was performed

We apologize for the mis-wording in our response. Indeed, "virulence" was not appropriate as the results were obtained during an *in vitro* experiment and we should have rather used viral infectious titer instead.

Therefore, we would like to suggest, at least to properly complete the first point before full acceptance.

Referee #3:

I think the author has answered each comment carefully. However, the authors have not responded to the advantages of this method compared to similar Bacteria-free reverse genetics (Torii et al., Cell reports, 2021 and Amarilla et al., Nature commun., 2021). At present, it is not considered novel enough to be published in EMBO Reports.

We apologize if the reviewer's remark has been skipped. In comparison to “conventional” reverse genetics technologies used to produce infectious clones, infectious viruses generated by ISA can be obtained with no need of labored technical steps (i.e. in vitro synthesis of full-length 30 kb RNA transcripts, cloning and sub-cloning of full length or fragmented genomes in bacteria). Similarly, compared to closest techniques that required a circular polymerase extension reaction (CPER) step of DNA fragments (Torii et al., Cell reports, 2021 and Amarilla et al., Nature commun., 2021), recombination of the DNA fragments produced after PCR in the ISA method is directly performed *in cellula*, making it lighter in term of technical steps.

This paragraph was added in line 333 of the final version of the revised manuscript.

Dr. Julien Mélade
Unité des Virus Émergents
France

Dear Dr. Mélade,

I am very pleased to accept your manuscript for publication in the next available issue of EMBO reports. Thank you for your contribution to our journal.

At the end of this email I include important information about how to proceed. Please ensure that you take the time to read the information and complete and return the necessary forms to allow us to publish your manuscript as quickly as possible.

As part of the EMBO publication's Transparent Editorial Process, EMBO reports publishes online a Review Process File to accompany accepted manuscripts. As you are aware, this File will be published in conjunction with your paper and will include the referee reports, your point-by-point response and all pertinent correspondence relating to the manuscript.

If you do NOT want this File to be published, please inform the editorial office within 2 days, if you have not done so already, otherwise the File will be published by default [contact: emboreports@embo.org]. If you do opt out, the Review Process File link will point to the following statement: "No Review Process File is available with this article, as the authors have chosen not to make the review process public in this case." Please note that the author checklist will still be published even if you opt out of the transparent process.

Thank you again for your contribution to EMBO reports and congratulations on a successful publication. Please consider us again in the future for your most exciting work.

Yours sincerely,

Achim Breiling
Editor
EMBO Reports

****

THINGS TO DO NOW:

You will receive proofs by e-mail approximately 2-3 weeks after all relevant files have been sent to our Production Office; you should return your corrections within 2 days of receiving the proofs.

Please inform us if there is likely to be any difficulty in reaching you at the above address at that time. Failure to meet our deadlines may result in a delay of publication, or publication without your corrections.

All further communications concerning your paper should quote reference number EMBOR-2021-53820V3 and be addressed to emboreports@wiley.com.

Should you be planning a Press Release on your article, please get in contact with emboreports@wiley.com as early as possible, in order to coordinate publication and release dates.

EMBO PRESS

YOU MUST COMPLETE ALL CELLS WITH A PINK BACKGROUND

PLEASE NOTE THAT THIS CHECKLIST WILL BE PUBLISHED ALONGSIDE YOUR PAPER

Corresponding Author Name: Julien Mélade
Journal Submitted to: EMBO reports
Manuscript Number: EMBOR-2021-53820V2

Reporting Checklist For Life Sciences Articles (Rev. June 2017)

This checklist is used to ensure good reporting standards and to improve the reproducibility of published results. These guidelines are consistent with the Principles and Guidelines for Reporting Preclinical Research issued by the NIH in 2014. Please follow the journal's authorship guidelines in preparing your manuscript.

A- Figures
1. Data
The data shown in figures should satisfy the following conditions:

- the data were obtained and processed according to the field's best practice and are presented to reflect the results of the experiments in an accurate and unbiased manner.
- figure panels include only data points, measurements or observations that can be compared to each other in a scientifically meaningful way.
- graphs include clearly labeled error bars for independent experiments and sample sizes. Unless justified, error bars should not be shown for technical replicates.
- if n< 5, the individual data points from each experiment should be plotted and any statistical test employed should be justified
- Source Data should be included to report the data underlying graphs. Please follow the guidelines set out in the author ship guidelines on Data Presentation.

2. Captions

Each figure caption should contain the following information, for each panel where they are relevant:

- a specification of the experimental system investigated (eg cell line, species name).
- the assay(s) and method(s) used to carry out the reported observations and measurements
- an explicit mention of the biological and chemical entity(ies) that are being measured.
- an explicit mention of the biological and chemical entity(ies) that are altered/varied/perturbed in a controlled manner.

- the exact sample size (n) for each experimental group/condition, given as a number, not a range;
- a description of the sample collection allowing the reader to understand whether the samples represent technical or biological replicates (including how many animals, litters, cultures, etc.).
- a statement of how many times the experiment shown was independently replicated in the laboratory.
- definitions of statistical methods and measures:
  - common tests, such as t-test (please specify whether paired vs. unpaired), simple χ2 tests, Wilcoxon and Mann-Whitney tests, can be unambiguously identified by name only, but more complex techniques should be described in the methods section;
  - are tests one-sided or two-sided?
  - are there adjustments for multiple comparisons?
  - exact statistical test results, e.g., P values = x but not P values < x;
  - definition of 'center values' as median or average;
  - definition of error bars as s.d. or s.e.m.

Any descriptions too long for the figure legend should be included in the methods section and/or with the source data.

In the pink boxes below, please ensure that the answers to the following questions are reported in the manuscript itself. Every question should be answered. If the question is not relevant to your research, please write NA (non applicable). We encourage you to include a specific subsection in the methods section for statistics, reagents, animal models and human subjects.

USEFUL LINKS FOR COMPLETING THIS FORM

http://www.antibodypedia.com
http://1degreebio.org
http://www.equator-network.org/reporting-guidelines/improving-bioscience-research-repor
http://grants.nih.gov/grants/olaw/olaw.htm
http://www.mrc.ac.uk/Ourresearch/Ethicsresearchguidance/Useofanimals/index.htm
http://ClinicalTrials.gov
http://www.consort-statement.org
http://www.consort-statement.org/checklists/view/32-consort/66-title
http://www.equator-network.org/reporting-guidelines/reporting-recommendations-for-tumo
http://datadryad.org
http://figshare.com
http://www.ncbi.nlm.nih.gov/gap
http://www.ebi.ac.uk/ega
http://biomodels.net/
http://biomodels.net/miriam/
http://jjj.biochem.sun.ac.za
https://osp.od.nih.gov/biosafety-biosecurity-and-emerging-biotechnology/
http://www.selectagents.gov/

B- Statistics and general methods

Please fill out these boxes (Do not worry if you cannot see all your text once you press return)

1.a. How was the sample size chosen to ensure adequate power to detect a pre-specified effect size?	For in vitro testing, we decided to performed experiment 3 times in order to obtain a mean value and look for the reproductibility of the assay
1.b. For animal studies, include a statement about sample size estimate even if no statistical methods were used.	The number of hamster used here were decided in regards to the article published previously in PMID: 33741945 and PMID: 32408338
2. Describe inclusion/exclusion criteria if samples or animals were excluded from the analysis. Were the criteria pre-established?	No animals or samples were excluded from the analysis. Every day, animals were weighed and monitored for the duration of the study to detect the appearance of any clinical signs of illness/suffering
3. Were any steps taken to minimize the effects of subjective bias when allocating animals/samples to treatment (e.g. randomization procedure)? If yes, please describe.	No steps were taken to minimize the subjective bias to treatment
For animal studies, include a statement about randomization even if no randomization was used.	No randomization was performed
4.a. Were any steps taken to minimize the effects of subjective bias during group allocation or/and when assessing results (e.g. blinding of the investigator)? If yes please describe.	No steps were taken to minimize the subjective bias during allocation or/and when asssssing results
4.b. For animal studies, include a statement about blinding even if no blinding was done	No blinding was done
5. For every figure, are statistical tests justified as appropriate?	Yes, statistical analysis are the same as used in previous pulished article PMID: 33741945
Do the data meet the assumptions of the tests (e.g., normal distribution)? Describe any methods used to assess it.	Exploratory analysis was performed using a two-way ANOVA with a Sidak's test correction. Statistical analysis and graphical representation were performed using GraphPad Prism 7.00. P values ≤ 0.05 were considered statistically significant.
Is there an estimate of variation within each group of data?	No estimation of variation

Is the variance similar between the groups that are being statistically compared?	Yes statistical comparaisons were performed in the same way between the groups

C- Reagents

6. To show that antibodies were profiled for use in the system under study (assay and species), provide a citation, catalog number and/or clone number, supplementary information or reference to an antibody validation profile. e.g., Antibodypedia (see link list at top right), 1DegreeBio (see link list at top right).	No antibodies were purchased
7. Identify the source of cell lines and report if they were recently authenticated (e.g., by STR profiling) and tested for mycoplasma contamination.	Cell lines were provided by ATCC; BHK-21; ATCC CCL-10; ATCC CRL-1586; AK-D; ATCC CCL-150

* for all hyperlinks, please see the table at the top right of the document

D- Animal Models

8. Report species, strain, gender, age of animals and genetic modification status where applicable. Please detail housing and husbandry conditions and the source of animals.	Syrian hamsters ; Female; aged three-weeks-old
9. For experiments involving live vertebrates, include a statement of compliance with ethical regulations and identify the committee(s) approving the experiments.	All experiments were approved by the local ethical committee (C2EA—14) and the French 'Ministère de l'Enseignement Supérieur, de la Recherche et de l'Innovation' (APAFIS#23975) and performed in accordance with the French national guidelines and the European legislation covering the use of animals for scientific purposes.
10. We recommend consulting the ARRIVE guidelines (see link list at top right) (PLoS Biol. 8(6), e1000412, 2010) to ensure that other relevant aspects of animal studies are adequately reported. See author guidelines, under 'Reporting Guidelines'. See also: NIH (see link list at top right) and MRC (see link list at top right) recommendations. Please confirm compliance.	We confirm compliance

E- Human Subjects

11. Identify the committee(s) approving the study protocol.	Hospital Clinical Research Program 2020-A01653-36, funded by the Assistance Publique - Hôpitaux de Marseille and approved by the Ethics Committee of South-West and Overseas France II
12. Include a statement confirming that informed consent was obtained from all subjects and that the experiments conformed to the principles set out in the WMA Declaration of Helsinki and the Department of Health and Human Services Belmont Report.	Twenty-four human sera were tested for the presence of SARS-CoV-2 neutralizing antibodies (nAbs). These samples were surplus to requirements from a SARS-CoV-2 research program (Hospital Clinical Research Program 2020-A01653-36, funded by the Assistance Publique - Hôpitaux de Marseille and approved by the Ethics Committee of South-West and Overseas France II) where participants provided written consent from research on SARS-COV-2 humoral immunity.
13. For publication of patient photos, include a statement confirming that consent to publish was obtained.	No patient photos was include
14. Report any restrictions on the availability (and/or on the use) of human data or samples.	No restrictions on the availability of human data and samples presented in this study
15. Report the clinical trial registration number (at ClinicalTrials.gov or equivalent), where applicable.	No clinical trial registration number was used
16. For phase II and III randomized controlled trials, please refer to the CONSORT flow diagram (see link list at top right) and submit the CONSORT checklist (see link list at top right) with your submission. See author guidelines, under 'Reporting Guidelines'. Please confirm you have submitted this list.	No phase II and phase III present in this study
17. For tumor marker prognostic studies, we recommend that you follow the REMARK reporting guidelines (see link list at top right). See author guidelines, under 'Reporting Guidelines'. Please confirm you have followed these guidelines.	No tumor marker prognostic studies present in this study

F- Data Accessibility

18: Provide a "Data Availability" section at the end of the Materials & Methods, listing the accession codes for data generated in this study and deposited in a public database (e.g. RNA-Seq data: Gene Expression Omnibus GSE39462, Proteomics data: PRIDE PXD000208 etc.) Please refer to our author guidelines for 'Data Deposition'.<br><br>Data deposition in a public repository is mandatory for:<br>a. Protein, DNA and RNA sequences<br>b. Macromolecular structures<br>c. Crystallographic data for small molecules<br>d. Functional genomics data<br>e. Proteomics and molecular interactions	SARS-CoV-2 European strain was obtained by the European Archive Collection (human isolate BetaCoV/Germany/BavPat1/2020 p.1; reference: 026V-03883)<br>FeCoV was obtained from the American Type Culture Collection (ATCC reference: VR-2126)<br>Wuhan SARS-CoV-2 genome Genbank acession number: NC045512<br>Coding sequence of the red fluorescent protein (mCherry) Genbank accession number: AY678264
19. Deposition is strongly recommended for any datasets that are central and integral to the study; please consider the journal's data policy. If no structured public repository exists for a given data type, we encourage the provision of datasets in the manuscript as a Supplementary Document (see author guidelines under 'Expanded View' or in unstructured repositories such as Dryad (see link list at top right) or Figshare (see link list at top right).	All datasets are available on appendix tables and expanded figures
20. Access to human clinical and genomic datasets should be provided with as few restrictions as possible while respecting ethical obligations to the patients and relevant medical and legal issues. If practically possible and compatible with the individual consent agreement used in the study, such data should be deposited in one of the major public access-controlled repositories such as dbGAP (see link list at top right) or EGA (see link list at top right).	No human clinical and genomics datasets was used here
21. Computational models that are central and integral to a study should be shared without restrictions and provided in a machine-readable form. The relevant accession numbers or links should be provided. When possible, standardized format (SBML, CellML) should be used instead of scripts (e.g. MATLAB). Authors are strongly encouraged to follow the MIRIAM guidelines (see link list at top right) and deposit their model in a public database such as Biomodels (see link list at top right) or JWS Online (see link list at top right). If computer source code is provided with the paper, it should be deposited in a public repository or included in supplementary information.	No computational models was used here

G- Dual use research of concern

22. Could your study fall under dual use research restrictions? Please check biosecurity documents (see link list at top right) and list of select agents and toxins (APHIS/CDC) (see link list at top right). According to our biosecurity guidelines, provide a statement only if it could.	The Dual Use Research of Concern (DURC) of this study have been carefully considered by our institution. All works were approved by the local committee and the French 'Ministère de l'Enseignement Supérieur, de la Recherche et de l'Innovation' with appropriate agreements described in the methods section.
